# Targeting TNF-**α**–producing macrophages activates antitumor immunity in pancreatic cancer via IL-33 signaling

**DOI:** 10.1172/jci.insight.153242

**Published:** 2022-11-22

**Authors:** Ajay Dixit, Aaron Sarver, Jon Zettervall, Huocong Huang, Kexin Zheng, Rolf A. Brekken, Paolo P. Provenzano

**Affiliations:** 1 Department of Biomedical Engineering, University of Minnesota, Minneapolis, Minnesota, USA.; 2 University of Minnesota Physical Sciences in Oncology Center, Minneapolis, Minnesota, USA.; 3 Masonic Cancer Center, University of Minnesota, Minneapolis, Minnesota, USA.; 4 Hamon Center for Therapeutic Oncology Research and Department of Surgery, UT Southwestern Medical Center, Dallas, Texas, USA.; 5 Department of Hematology, Oncology, and Transplantation;; 6 Institute for Engineering in Medicine;; 7 Stem Cell Institute; and; 8 Center for Multiparametric Imaging of Tumor Immune Microenvironments, University of Minnesota, Minneapolis, Minnesota, USA.

**Keywords:** Oncology, Cancer immunotherapy, Cellular immune response, Gastric cancer

## Abstract

Pancreatic ductal adenocarcinoma (PDA) remains resistant to immune therapies, largely owing to robustly fibrotic and immunosuppressive tumor microenvironments. It has been postulated that excessive accumulation of immunosuppressive myeloid cells influences immunotherapy resistance, and recent studies targeting macrophages in combination with checkpoint blockade have demonstrated promising preclinical results. Yet our understanding of tumor-associated macrophage (TAM) function, complexity, and diversity in PDA remains limited. Our analysis reveals significant macrophage heterogeneity, with bone marrow–derived monocytes serving as the primary source for immunosuppressive TAMs. These cells also serve as a primary source of TNF-α, which suppresses expression of the alarmin IL-33 in carcinoma cells. Deletion of *Ccr2* in genetically engineered mice decreased monocyte recruitment, resulting in profoundly decreased TNF-α and increased IL-33 expression, decreased metastasis, and increased survival. Moreover, intervention studies targeting CCR2 with a new orthosteric inhibitor (CCX598) rendered PDA susceptible to checkpoint blockade, resulting in reduced metastatic burden and increased survival. Our data indicate that this shift in antitumor immunity is influenced by increased levels of IL-33, which increases dendritic cell and cytotoxic T cell activity. These data demonstrate that interventions to disrupt infiltration of immunosuppressive macrophages, or their signaling, have the potential to overcome barriers to effective immunotherapeutics for PDA.

## Graphical abstract

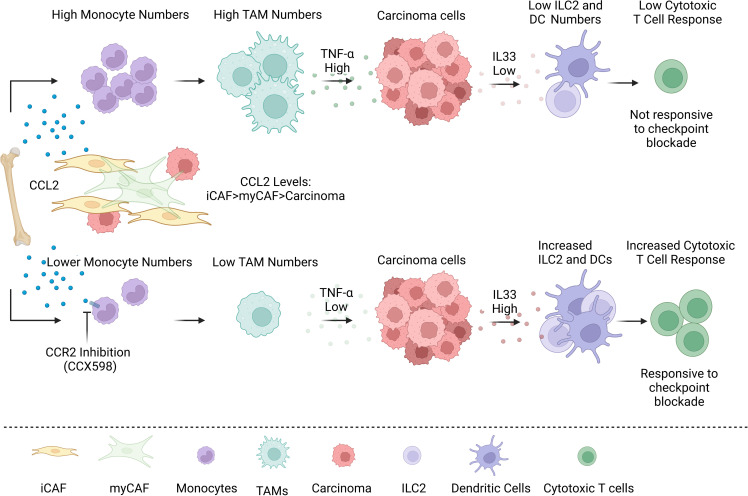

## Introduction

Pancreatic ductal adenocarcinoma (PDA) is one of the most lethal forms of cancer (1, 2). This is due, in part, to robust metastatic behavior and multiple mechanisms of resistance to molecular, immune, and radiation therapy interventions (3, 4). Importantly, PDA is characterized by a robust stromal fibrotic and immunosuppressive response that creates drug-free and antitumor immunity–free sanctuaries in primary and metastatic disease (5–9). While immune therapy with immune checkpoint blockade (ICB) has been successful in rare cases (10), most pancreatic cancers are resistant to ICB (11). Yet, overcoming stromal barriers found in PDA can render the disease susceptible to ICB (12–16), demonstrating that a robust antitumor immune response can take place in PDA under the right therapeutic conditions.

In addition to dense extracellular matrix (ECM) and the immunosuppressive behavior of cancer-associated fibroblasts (CAFs), one of the primary barriers to an effective antitumor immune response in PDA is thought to be the abundance and activity of immunosuppressive tumor-associated macrophage (TAM) populations (16–19). Indeed, myeloid-derived suppressor cells (MDSCs) and macrophages are often the most abundant stromal populations in PDA (14, 20). This has strong implications for disease progression and resistance to therapy, since distinctly polarized macrophages are capable of promoting all steps of tumor progression, including carcinoma cell proliferation, invasion, and colonization of metastatic sites, as well as having robust inflammatory and immunosuppressive functions (21–24). Furthermore, in addition to a spectrum of polarization states, distinct behaviors can emerge from tissue-resident macrophages versus infiltrating macrophages that are derived from bone marrow progenitor cells, where the latter are thought to play a larger role in immune regulation (23, 24). In this context, macrophages can not only prevent efficient T cell infiltration into the tumor (17, 24) but also decrease the ability of intratumoral T cells to recognize and kill tumor cells (23, 25, 26), ultimately posing a major obstacle to effective immunotherapy. Given their importance in tumor dynamics, therapeutic approaches that either deplete or reprogram macrophages have been proposed in order to improve immune therapy outcomes. However, a comprehensive characterization of macrophage heterogeneity and the mechanisms that cause a weak antitumor immune response in PDA still remains elusive. In this study using single-cell RNA sequencing (scRNA-Seq) data, we further define the macrophage landscape of murine and human PDA. We identify unique and targetable features of infiltrated immunosuppressive macrophages that are derived from bone marrow progenitor cells. Notably, monocytes utilize CCR2 to infiltrate into PDA, where CCL2 is secreted by carcinoma cells and to a larger degree by multiple CAF phenotypes. Notably, genetic depletion of CCR2 in genetically engineered murine models of PDA results in a reduction of infiltrated macrophages, leading to decreased metastasis and increased survival. Consistent with these findings, focused inhibition of CCR2 with a novel inhibitor renders primary and metastatic PDA tumors susceptible to ICB, a behavior that is augmented, in part, through increased antitumor immunity from elevated levels of IL-33 in cancer cells. Mechanistically, TNF-α, predominantly secreted by TAMs, decreases the expression of the alarmin IL-33 in carcinoma cells. Once IL-33 is released, it helps regulate the attraction of CD103^+^ dendritic cells and ultimately a more robust cytotoxic T cell response. Thus, these data collectively expand our knowledge of TAM dynamics in PDA and present an effective strategy to reduce immunosuppression to achieve effective antitumor immunity.

## Results

### Diverse TAMs coexist in human PDA.

**Figure 1:**
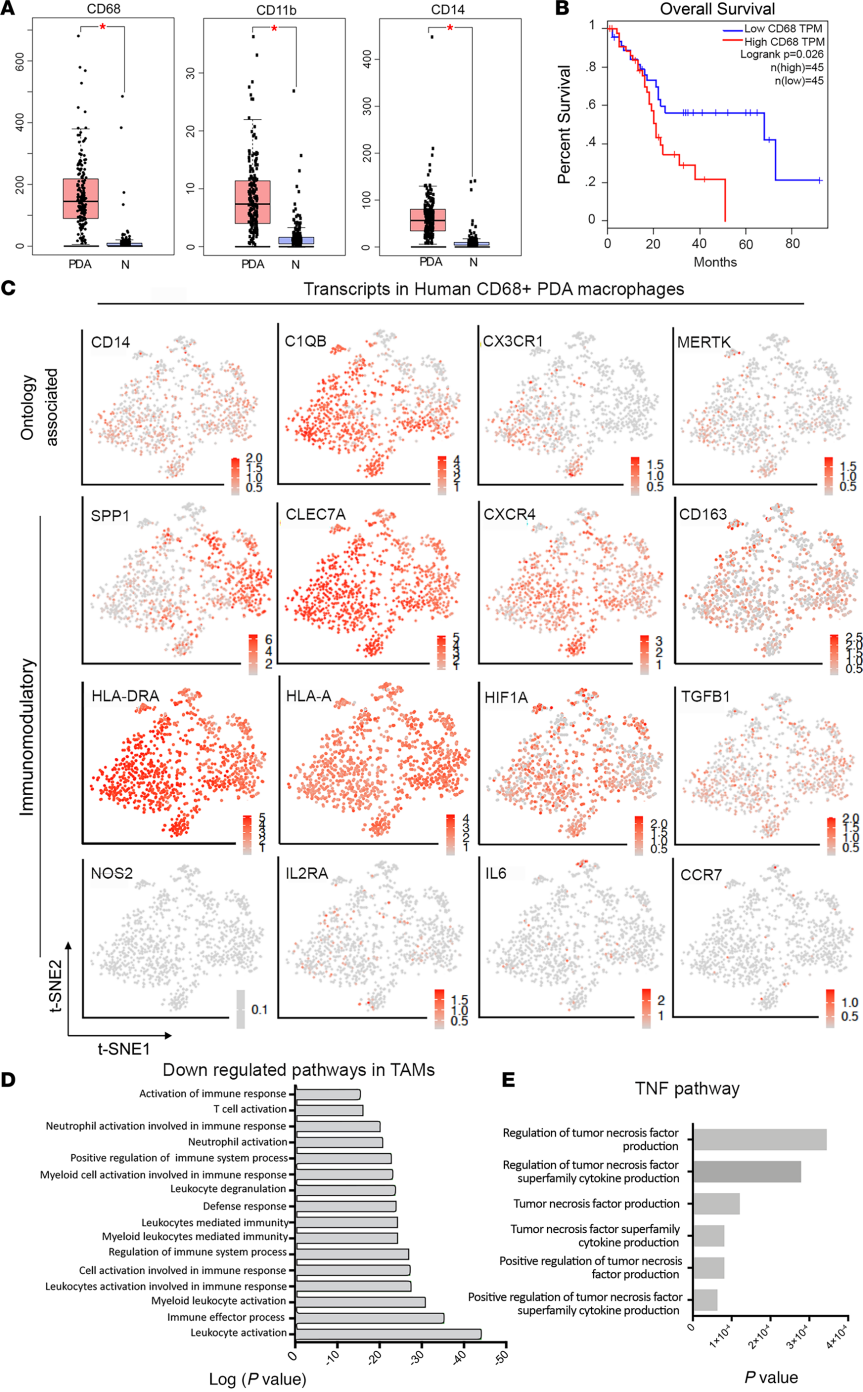
Human PDA has heterogeneous macrophage populations. (**A**) TCGA data set analysis demonstrating that transcripts identifying myeloid cells and TAMs in PDA (i.e., CD68, CD11b, and CD14) are overexpressed in PDA. *P < 0.0001. (**B**) Transcripts associated with CD68^+^ macrophages correlate with poor prognosis in PDA patients (TCGA data set analysis). TPM, transcripts per million. (**C**) Distribution of factors that regulate macrophage polarization and function within the heterogeneous macrophage populations in PDA showing the strongest signal for immunosuppressive factors. (**D** and **E**) Gene Ontology pathway analysis of human PDA CD68^+^ macrophages showing decreased antitumor immune activation (i.e., immunosuppressive behavior) (**D**) and increased pathways enriched in TNF-α production (**E**).

Analysis of transcriptome data demonstrated that human PDA is characterized by excessive accumulation of heterogeneous macrophages (Figure 1A and Supplemental Figure 1A; supplemental material available online with this article; https://doi.org/10.1172/jci.insight.153242DS1), consistent with previous reports (14, 20). This macrophage expansion and infiltration starts at the early stages of disease (8, 20) (Supplemental Figure 1B), and increased levels of macrophages correlated with poor prognosis in PDA patients (Figure 1B). To further characterize human PDA macrophage populations, we used scRNA-Seq data from human PDA patients (*n* = 6) and 2 cancer-adjacent normal pancreas samples (*n* = 2) (data from ref. 27, where it is noted that more than 96% of cells in this grouping were identified as monocytes or macrophages). Overall, all the macrophages showed patient-specific heterogeneity (Supplemental Figure 1C). We observed distinct clusters and distribution of genes associated with processes such as proinflammation, matrix remodeling, metabolism, and immunosuppression using dimensionality reduction with t-distributed stochastic neighbor embedding. Overall, we found that, consistent with other reports (23, 28), PDA TAMs displayed a more alternatively activated (M2-like) polarization with the expression of genes associated with the M2 phenotype and immunosuppression such as *SPP1*, *CD163*, *CXCR4*, *HIF1*, *TGF-β1*, and multiple MHCII molecules (Figure 1C). Likewise, the vast majority of PDA TAMs robustly expressed the M2 marker CLEC*7A* (*Dectin-1*), which in concert with its ligand galectin 9 promotes strong immune suppression from macrophages that can be blocked to promote antitumor immunity in PDA (29). Further, PDA TAMs displayed shifts in metabolism (Supplemental Figure 1D). The majority of TAMs displayed a heterogeneous expression of transcripts associated with higher immunosuppressive metabolism and signaling such as *KYNU* (tryptophan metabolism), *NLRP3* (inflammasome/IL-18), *ADK* (adenosine metabolism), and *STAT3* (Supplemental Figure 1D). Notably, tryptophan production and kynurenine production are known to inhibit T cell proliferation and cytotoxic activity (30). Likewise, TAMs also expressed high levels of ADK, which is involved in adenosine metabolism, where adenosine metabolites promote antiinflammatory macrophage phenotype. In contrast, transcripts suggesting a classically activated M1-like polarization such as *CCR7*, *IL-6*, and *IL-2RA* were low, while *NOS2* (iNOS), a robust M1 marker, was extremely low (Figure 1C), further supporting the conclusion that the majority of TAMs in PDA possess a more alternatively activated phenotype. Collectively, this culminated with strong statistical enrichment for immunosuppressive TAM function (Figure 1D) and also high enrichment for TNF-α production pathways (Figure 1E), which is consistent with high TNF-α levels in the PDA stroma (Supplemental Figure 1E), further suggesting that PDA TAMs could be a major source of TNF-α in PDA and are predominantly immunosuppressive.

Further analysis of the human PDA macrophage transcriptome supports findings from mice demonstrating that macrophages are either tissue resident or bone marrow derived (20, 27); pancreatic resident macrophages originate from the fetally derived yolk sac and are maintained independently of circulating macrophages, while infiltrating macrophages arise from circulating CD14^+^ monocytes. Indeed, PDA TAMs are a mixed population displaying markers of both populations. However, the majority of TAMs across all patients displayed high gene expression of *CD14*, *C1QB*, and MHCII (e.g., *HLA-DR*; Figure 1C) typically associated with bone marrow–derived macrophages (20, 28). For instance, about 80% of TAMs that originate from infiltrated hematopoietic stem cell–derived monocytes are MHCII^hi^ (20), and monocytes that infiltrate tissue and differentiate into macrophages can be distinguished by high expression of C1QB (31) (Figure 1C). In contrast, genes associated with resident macrophages such as *CX3CR1* or *MerTK* were also expressed but were noticeably less abundant, suggesting that despite their heterogeneity the majority of TAMs originated from bone marrow.

### Immunosuppressive TAMs in genetically engineered murine models of PDA.

**Figure 2:**
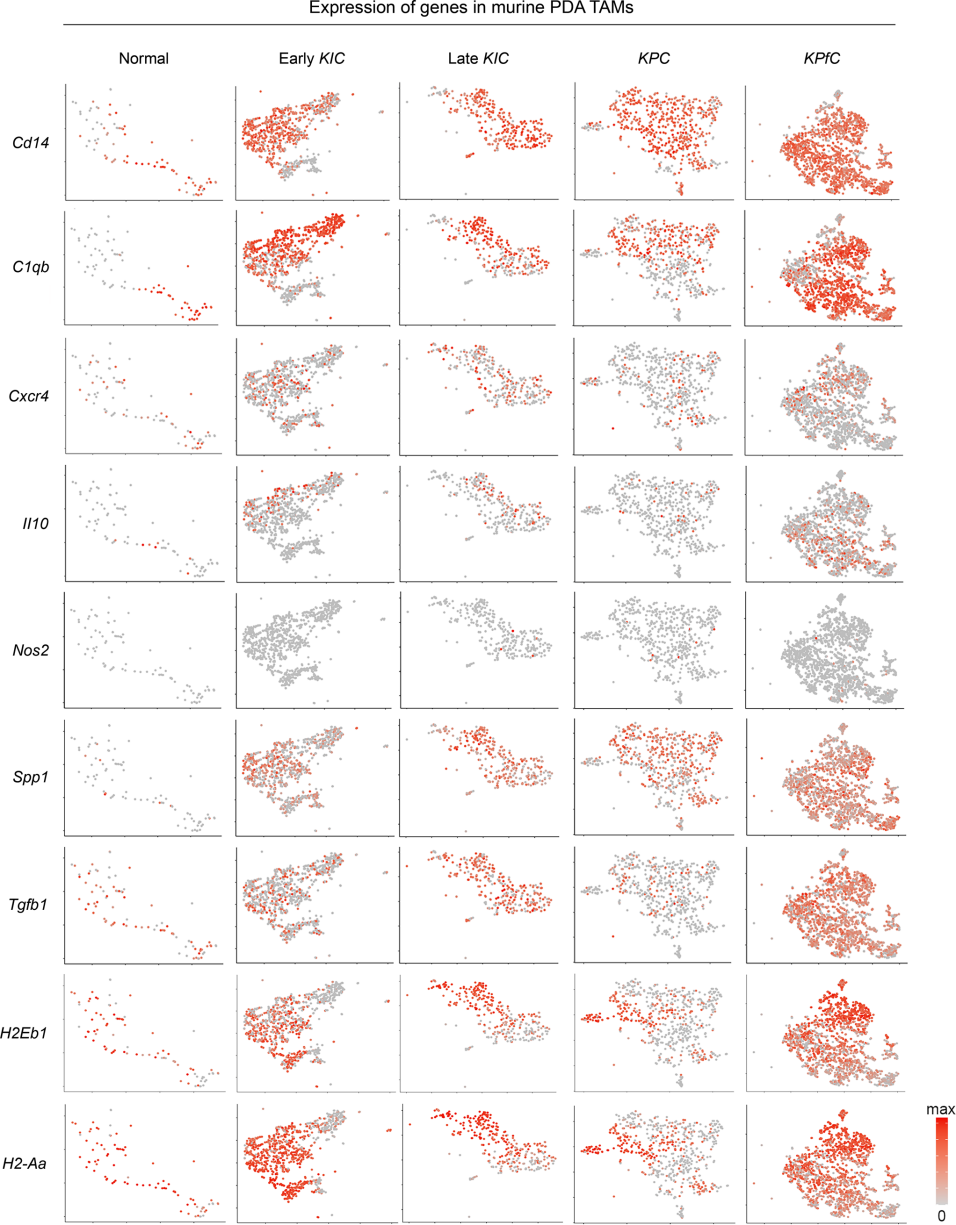
Cross-species examination of macrophages from *KPC*, *KIC*, and *KPfC* genetically engineered murine models of PDA demonstrates similarities to human macrophages. Visualization of transcript distributions in murine macrophage populations using t-distributed stochastic neighbor embedding (t-SNE) shows that expression patterns in *KPC*, *KIC*, and *KPfC* macrophages, like those in human PDA macrophages, suggest robust immunosuppressive behavior.

To examine how well human PDA TAMs are represented in various murine models of PDA, we extended our investigation to the analysis of scRNA-Seq data from 3 different genetically engineered murine models of PDA: the *KIC* (*Kras^LSL-G12D/+^ Ink4a^fl/fl^ Ptf1a^Cre/+^*), *KPC* (*Kras^LSL-G12D/+^ Trp53^LSL-R172H/+^ Ptf1a^Cre/+^*), and *KPfC* (*Kras^LSL-G12D/+^ Trp53^fl/fl^ Pdx1-Cre*) systems (Figure 2), as described previously (32). Overall, TAM populations observed in human PDA were also well represented in these murine models. Consistent with human data, murine PDA TAMs displayed high expression of M2-associated and immunosuppressive transcripts, such as *Spp1*, *C1qb*, *Arg1*, *Tgfb1*, and multiple MHCII molecules (e.g., *H2-Aa*, *H1-Ab1*, *H2-Dma*, *H2-Dmb1*, *H2-Dmb2*, and *H2-Eb1*; Figure 2 and Supplemental Figure 2). Furthermore, similar to human data, M1 markers (e.g., *Nos2*, *Ccr5*, *Il-6*) were found at a lower frequency (Supplemental Figure 2), consistent with previous data showing low levels of iNos^+^ TAMs in murine PDA (15). Further, *Cd14*-, *C1qb*-, and *Cxcr4*-expressing macrophages were present in lower numbers in the normal pancreas but expanded in early disease and were maintained throughout late-stage disease. Likewise, the presence of *Spp1*^+^ and *Arg1*^+^ macrophages was extremely low in normal pancreas, but their number increased in PDA. Together, these data demonstrate robust TAM heterogeneity in murine PDA similar to that seen in human PDA, with dominant immunosuppressive features and the majority showing high expression of markers indicating they are bone marrow–derived in origin.

### CCL2 is overexpressed by distinct cell populations at primary and metastasis sites.

**Figure 3:**
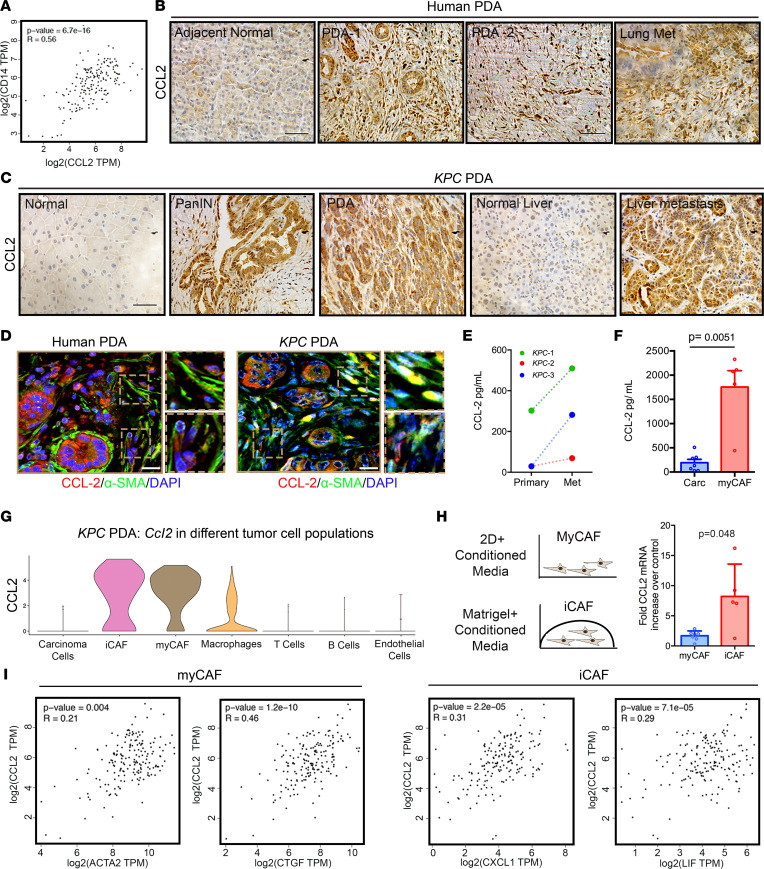
Both iCAFs and myCAFs secrete high levels of CCL2 in PDA tumor microenvironments. (**A**) Human TCGA data set analysis shows a strong correlation between expression levels of *CCL2* and *CD14*. (**B**) CCL2 is overexpressed in human PDA. IHC analysis demonstrates high expression of CCL2 in both human primary PDA tumors and metastatic lesion (lung). (**C**) CCL2 is also overexpressed in the genetically engineered *KPC* model of PDA at all stages. IHC shows high expression in PanINs, primary tumor, and metastatic lesions (liver). (**D**) CCL2 is colocalized with carcinoma cells and both α-SMA^lo^ and α-SMA^hi^ cells in the stroma of both human and murine PDA. Scale bars: 50 μm (**B**–**D**). (**E**) Metastatic cancer cells secrete higher levels of CCL2 than carcinoma cells derived from primary tumors (CCL2 levels were measured in culture supernatant by ELISA for paired primary and metastatic cell lines derived from *KPC* mice). (**F**) CAFs secrete higher CCL2 than carcinoma cells. CCL2 levels in culture supernatant of CAFs (grown on 2D culture plates with serum, i.e., myCAFs) and carcinoma cells were measured by ELISA (*n* = 5–7 *KPC* cell lines; *P* value was derived by Mann-Whitney test). (**G** and **H**) CAFs are the major CCL2 contributors in PDA. Violin plots of *Ccl2* transcripts for individual cell populations in *KPC* PDA show that iCAFs and myCAFs both express higher levels of *Ccl2* compared with other tumor cell populations. The expression of *Ccl2* in both myCAFs and iCAFs was validated experimentally by quantitative PCR (*n* = 5–7 *KPC* cell lines; *P* value was derived by Mann-Whitney *U* test). (**I**) Strong correlations between *CCL2* and both iCAF marker genes (*CXCL1*, *LIF*) and myCAF marker genes (*ACTA*, *CTGF*) in human TCGA data sets demonstrating that *CCL2* is highly expressed by both iCAFs and myCAFs.

Our data analysis in both human and murine PDAs suggests that the majority of immunosuppressive TAMs originate from bone marrow–derived monocytes. Loss or inhibition of CCR2 is known to significantly reduce levels of circulating monocytes and, as a result, numbers of bone marrow–derived TAMs (20, 33), and reducing TAMs may be beneficial as a therapeutic strategy against PDA (e.g., 14, 17, 18, 34). Therefore, to examine the expression of the CCR2 ligand CCL2 in PDA, we first performed correlation analysis with human patient data and observed a significant correlation between *CCL2* and *CD14* (Figure 3A), as well as other macrophage markers such as *CXCR4*, *CD206*, *CD163*, and MHCII transcripts (not shown), further linking expression of CCL2 in tumors with myeloid cell infiltration. Furthermore, examination of CCL2 levels using IHC demonstrated overexpression in the primary tumor as well as in a metastatic lesion of human PDA (Figure 3B), where *CCL2* was overexpressed starting in early disease and continued to be expressed highly throughout disease progression (Supplemental Figure 3A). Similarly, elevated expression was seen in pancreatic intraepithelial neoplasia (PanIN) and primary and metastatic PDA in *KPC* mouse samples (Figure 3C). In murine and human PDA, IHC revealed CCL2 expression in carcinoma cells and the stromal compartment (Figure 3, B and C), suggesting that multiple cell populations in PDA can recruit circulating monocytes. To confirm this, we performed immunofluorescent staining for CCL2 and α-SMA, a marker for a dominant subtype of carcinoma-associated fibroblasts, or myofibroblastic CAFs (myCAFs), in human PDA, and observed CCL2 localization with carcinoma cells, α-SMA^+^ CAFs, and α-SMA^lo^ or α-SMA^–^ cells in the stroma (Figure 3D). Therefore, we sought to further evaluate the relative CCL2 contribution of carcinoma cells and CAFs. Interestingly, while primary carcinoma cells secreted CCL2, matched metastatic lines (i.e., lines from metastatic lesions in the same animals) showed greater mRNA (Supplemental Figure 3B) and CCL2 secretion (Figure 3E). Yet primary CAFs from *KPC* tumors secreted substantially higher CCL2 protein when compared with carcinoma cells (Figure 3F).

As recent studies have highlighted CAF heterogeneity in PDA (27, 32, 35, 36), we further dissected CCL2 secretion from 2 prominent CAF populations, myCAFs and inflammatory CAF (iCAFs), that we generated from *KPC* tumors as described previously (35). Remarkably, both CAF phenotypes showed significantly higher expression of CCL2 compared with primary or metastatic carcinoma cells, which is consistent with scRNA-Seq data and moderate correlations between iCAF or myCAF transcript markers and *CCL2* in human PDA (Figure 3I). However, iCAFs expressed significantly higher levels of CCL2 than myCAFs (Figure 3H and Supplemental Figure 3D), suggesting a hierarchy for recruiting monocyte-derived macrophages to immunosuppressive niches in order of iCAFs > myCAFs > metastatic cells > primary carcinoma cells. However, it is notable that both iCAFs and myCAFs expressed profoundly more CCL2 cytokine than other cell populations from PDA tumors (Figure 3, F–H, and Supplemental Figure 2D), suggesting they both have a robust capacity to recruit monocytes via CCL2. In fact, this behavior was observed for a number of factors known to promote PDA progression and/or therapeutic resistance (e.g., collagens, proteoglycans, hyaluronan synthesis, lysyl oxidase, IGF-1, IL-6, CSF1, etc.; Supplemental Figure 4), while others, such as CXCL12, appeared more phenotype specific. Thus, compared with the broader cell populations in PDA (vs. comparing levels solely between iCAFs and myCAFs), it is clear that many key factors are elevated in both iCAFs and myCAFs relative to other cell types but sometimes to different degrees (i.e., both are significant sources of key ECM proteins, cytokines, and growth factors), adding further complexity to dissecting distinct roles of CAF phenotype, particularly spatially and temporally within complex tumor microenvironments.

### Ccr2 deletion delays PDA progression and reduces metastasis to improve overall survival.

**Figure 4:**
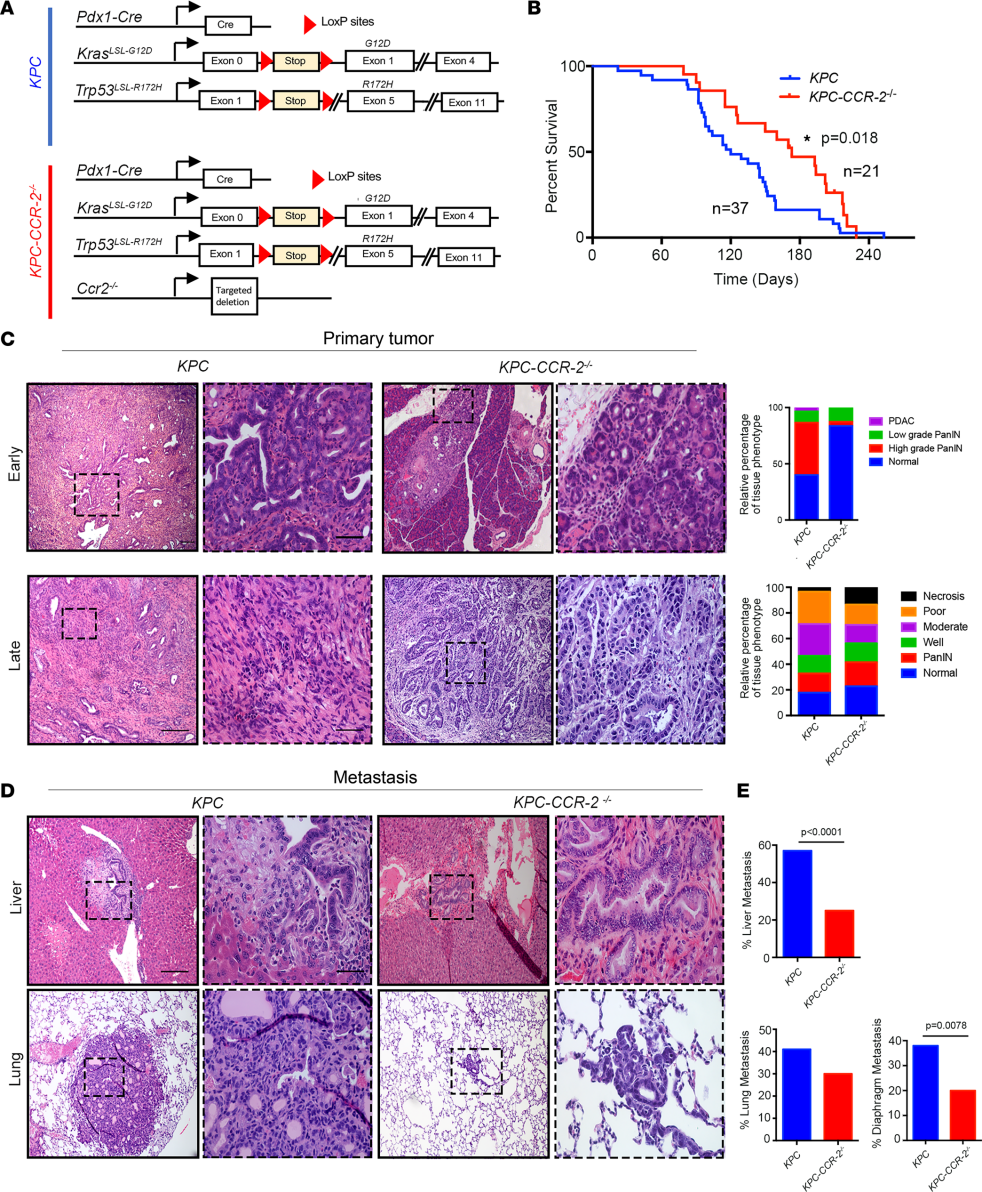
Genetic deletion of *Ccr2* reduces disease severity. (**A**) Schematics of the *KPC* mouse model of pancreatic cancer: *Kras^LSL-G12D/+^* *p53^LSL-R172H/+^* *Pdx1*-*Cre*. Deletion of *Ccr2* was attained by crossing of *KPC* with *Ccr2^–/–^* (global) mice, referred to as *KPC-CCR2^–/–^*. (**B**) Kaplan-Meier analysis comparing survival of *KPC* (*n* = 37) and *KPC-CCR2^–/–^* (*n* = 21) mice demonstrates that *KPC-CCR2^–/–^* mice survive longer than *KPC* mice. *P* = 0.018 by log-rank test. (**C**) *KPC-CCR2^–/–^* animals show delayed PDA onset. Comparative H&E staining and histopathology of pancreata from *KPC* and *KPC-CCR2^–/–^* mice in early disease (10–11 weeks) (*n* = 3 in each group) and at the endpoint (*KPC*, *n* = 22; *KPC-CCR2^–/–^*, *n* = 19) show less advanced disease throughout the pancreas of *KPC-CCR2^–/–^* mice. (**D** and **E**) *KPC-CCR2^–/–^* mice show decreased metastasis. Scale bars: Early: 100 μm (50 μm for the boxed areas) and Late: 200 μm (50 μm for the boxed areas). (**D**) Representative H&E-stained images of liver and lung metastatic lesions from *KPC* and *KPC-CCR2^–/–^* cohorts. Scale bars: 200 μm (50 μm for the boxed areas). (**E**) Percentage of metastasis in various organs of *KPC* (*n* = 22) and *KPC-CCR2^–/–^* (*n* = 19) animals. *P* value by Fisher’s exact test.

To better understand the influence of CCL2/CCR2 signaling and the impact of bone marrow–derived TAMs in PDA, we generated *KPC* mice lacking *Ccr2* genes. *Ccr2*-deleted mice have been reported to have reduced capacity for monocyte recruitment from bone marrow (37). To generate *KPC*-*CCR2–*knockout mice, *KC* mice (*Kras^LSL-G12D/+^ Pdx1-Cre*) were crossed with mice with global knockout of *Ccr2* and then bred with *Trp53^LSL-R172H/LSL-R172H^* mice (Figure 4A). The progeny were born in the expected Mendelian ratio, with no obvious functional defects. The deletion of *Ccr2* in *KPC-CCR2^–/–^* was confirmed by PCR (Supplemental Figure 5A). As expected, the loss of CCR2 led to a profound reduction of circulating CD11b^+^ myeloid cells (>80% reduction; Supplemental Figure 5B), which resulted in a concomitant decrease in the PDA stroma (Supplemental Figure 5C). Interestingly, examination of full survival data demonstrated that *KPC-CCR2^–/–^* mice survived significantly longer compared with *KPC* littermates (median survival 168 vs. 120 days, respectively; Figure 4B). Histological examination of pancreatic tumors from early-stage (10–11 weeks old) *KPC* and *KPC-CCR2^–/–^* mice showed that *KPC-CCR2^–/–^* mice had more disease-free normal pancreatic tissue and lower-grade PanINs than the *KPC* group (Figure 4C), consistent with the right-shifted survival curve (Figure 4B), suggesting a slower progression of disease. Consistent with this finding, depletion of circulating myeloid cells also reduced the proliferation of carcinoma cells in early- and late-stage disease (Supplemental Figure 5D). Examination of PDA in both cohorts showed regions of well-differentiated, moderately differentiated, poorly differentiated, and necrotic regions; however, *KPC-CCR2^–/–^* mice again showed less high-grade tumor as well as significantly less metastatic burden, but no differences in local invasion, α-SMA^+^ CAF frequency, or fibrillar collagen deposition, when compared with *KPC* mice (Figure 4C and Supplemental Figure 5, D–G), suggesting that reduced levels of CCL2-recruited TAMs in PDA slow disease progression and reduce metastatic burden, resulting in longer survival of *KPC* mice.

Examination of the metastatic distribution and burden in *KPC* mice demonstrated that while 91% of *KPC* mice displayed metastatic dissemination, only 40% of *KPC-CCR2^–/–^* presented with metastasis (Figure 4, D and E; Table 1; and Supplemental Table 1). In the liver, 57% of mice in the *KPC* cohort displayed metastatic lesions, in contrast to 25% in *KPC-CCR2^–/–^* mice (Figure 4E). *KPC-CCR2^–/–^* mice also exhibited a lower percentage of diaphragm metastasis (38% vs. 20%) and a nonsignificant trend of decreasing lung metastasis (41% vs. 30%). Last, we note that recent studies have established a role for elevated fibronectin (FN) in promoting the pre-metastatic niche, in part through the recruitment of bone marrow–derived macrophages (38, 39). Therefore, to determine whether our observed reduction in metastasis was due, at least in part, to a decrease in pre-metastatic niche formation, we measured the levels of FN in the 8- to 11-week-old mice. No differences in FN levels were observed in the liver or lungs (Supplemental Figure 5H), suggesting that the observed decrease was not due to a difference in key ECM in the pre-metastatic niche formation but rather could have been due to decreased macrophages. Taken together, these data suggest that the depletion of CCR2-mediated macrophage infiltration profoundly decreases metastatic disease.

### CCR2 inhibition mitigates the immunosuppressive environment and renders PDA susceptible to ICB.

**Figure 5:**
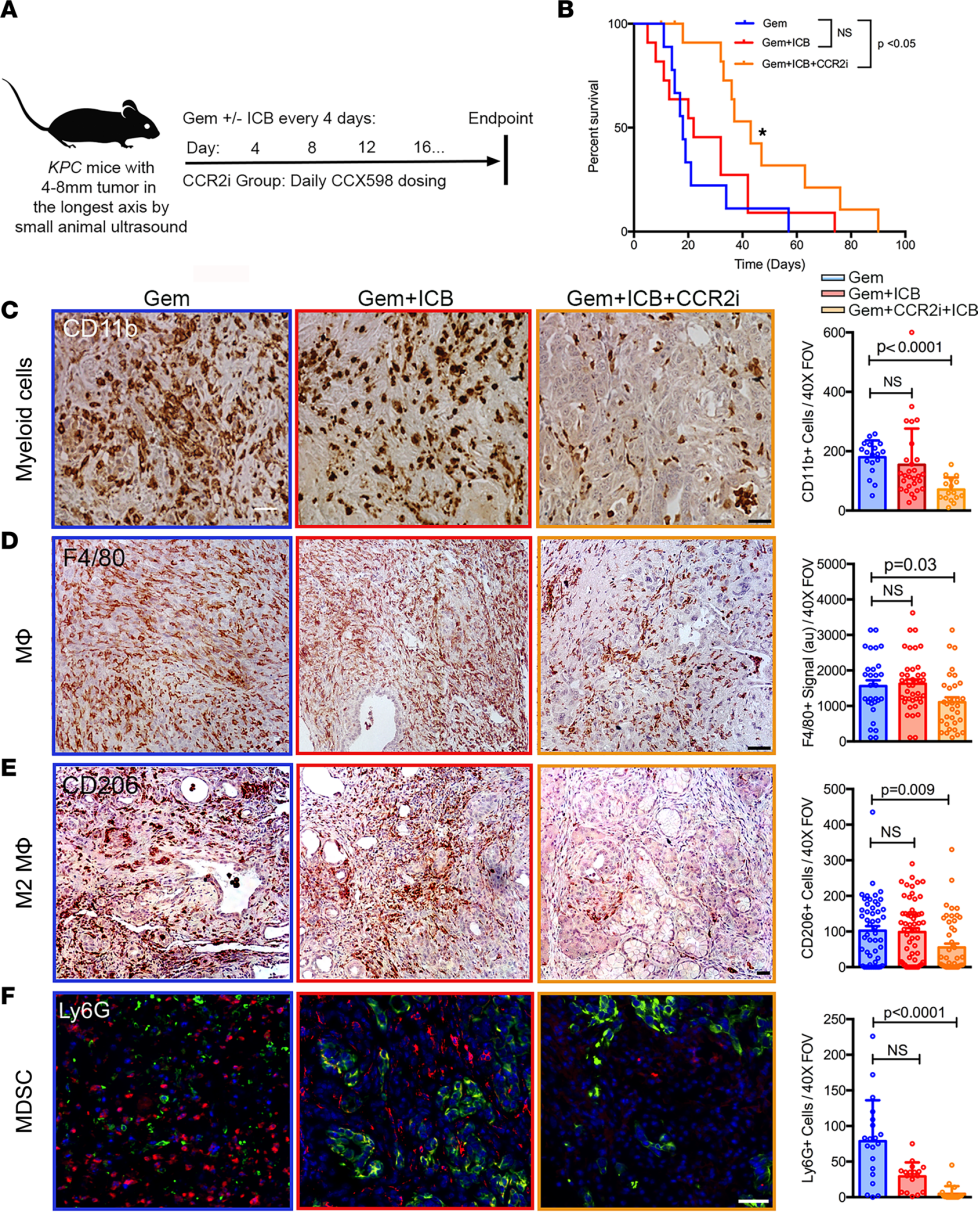
Blocking infiltration of bone marrow–derived TAMs increases responsiveness to immune therapy in *KPC* mice. (**A**) Schematic of the therapy regime. (**B**) Kaplan-Meier curve showing that Gem+ICB+CCR2i–treated animals have significantly longer survival compared with Gem and Gem+ICB cohorts. *P < 0.05. (**C**–**F**) IHC/IF analysis demonstrates that Gem+ICB+CCR2i combination therapy results in significant decreases in total CD11b^+^ myeloid cells (**C**), F4/80^+^ macrophages (**D**), CD206^+^ immunosuppressive macrophages (**E**), and MDSCs (**F**). *P* values by Kruskal-Wallis and Dunn’s multiple-comparison tests; *n* =4–6 animals in each group. Scale bars: 50 μm.

**Figure 6:**
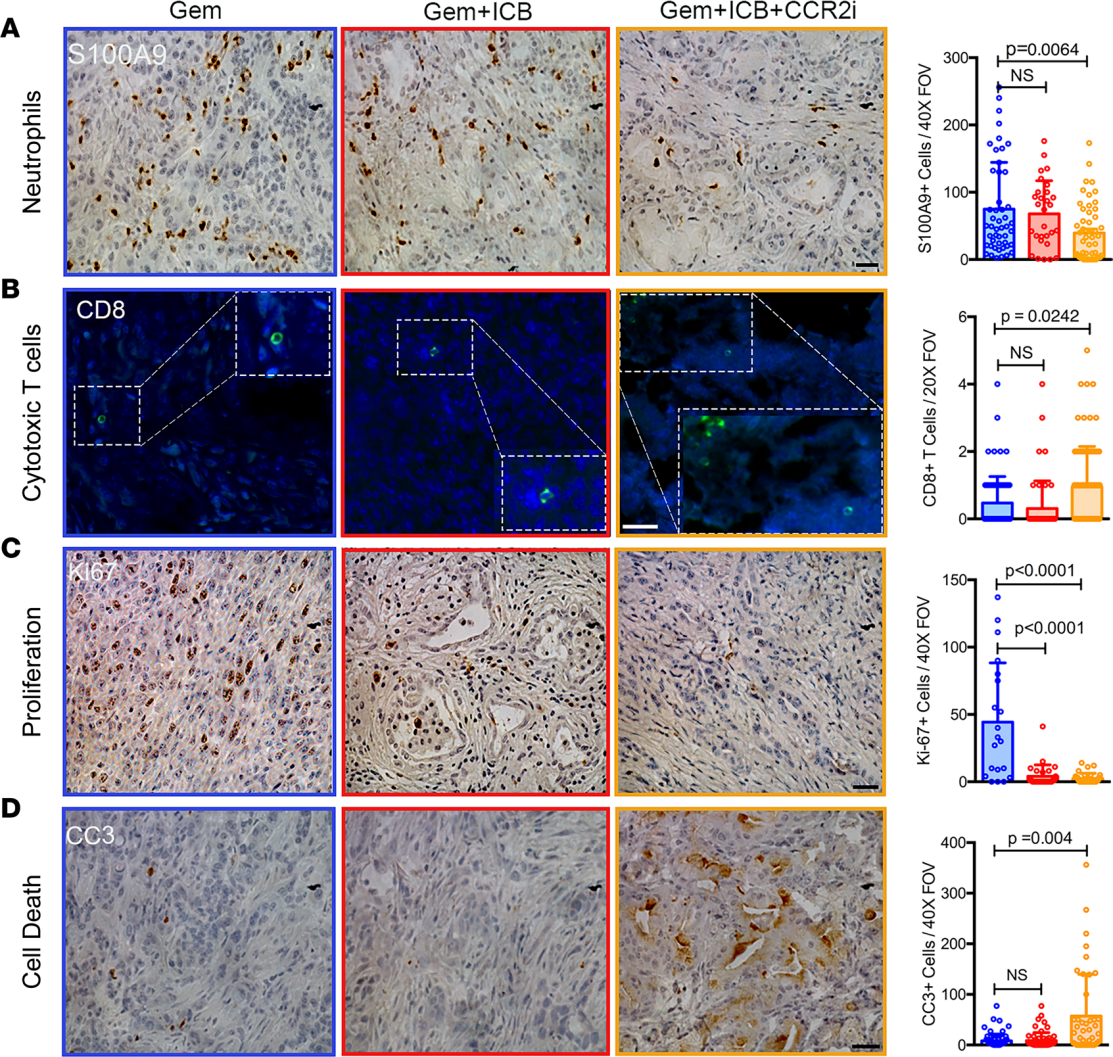
Blocking infiltration of bone marrow–derived TAMs increases cytotoxic T cell levels and carcinoma cell death in *KPC* mice. (**A**) IHC/IF analysis demonstrates that Gem+ICB+CCR2i combination therapy results in significant decreases in neutrophils within PDA. (**B**) IF staining shows significant increases in CD8^+^ cytotoxic T cells in the Gem+ICB+CCR2i treatment group. (**C**) Gem+ICB+CCR2i–treated animals have lower numbers of Ki67^+^ cells. (**D**) Gem+ICB+CCR2i therapy increases cell death as shown by IHC staining for cleaved caspase-3 (CC3^+^). Cell number or signal per field of view is shown. *P* values by Kruskal-Wallis and Dunn’s multiple-comparison tests; *n* = 4–6 animals in each group. The scale bar for the main images is 50 μm. The magnifications are 1.5×.

Given collective findings by us and others showing a predominant immunosuppressive TAM phenotype in PDA and our data from *KPC-CCR2^–/–^* mice, we sought to deplete bone marrow–derived monocytes to test the hypothesis that depletion of infiltrated TAMs is an avenue for the development of new combination immunotherapies for PDA. To test our hypothesis, we enrolled the *KPC* mice with 4 to 8 mm tumors in the longest axis by high-resolution small-animal ultrasound into treatment cohorts using a rolling enrollment model. The enrolled mice were treated with (a) standard-of-care chemotherapy with gemcitabine (Gem), (b) gemcitabine in combination with ICB in the form of anti–PD-1 and anti–CTLA-4 (Gem+ICB), or (c) a combination of gemcitabine, ICB, and CCR2 inhibition (Gem+ICB+CCR2i) (Figure 5A). To our knowledge, CCR2 inhibition in combination with ICB immune therapies has not been previously tested in PDA, especially using autochthonous disease that arises in the *KPC* model, and we focused on preclinical drug combinations that could be clinically viable (i.e., that include a standard-of-care chemotherapy). In order to inhibit CCR2, we tested a new orthosteric inhibitor, CCX598 (a potent third-generation CCR2 antagonist from ChemoCentryx). CCR2 inhibitor was given orally every day, a dosing scheme that achieves the desired concentration in the circulation to obtain receptor coverage (Supplemental Figure 6A). Importantly, and consistent with our hypothesis, the Gem+ICB+CCR2i combination therapy significantly increased the survival of *KPC* mice compared with Gem or Gem+ICB treatments (Figure 5B), while the addition of ICB did not improve outcomes from gemcitabine alone, consistent with other reports showing that without a stroma-targeting approach chemotherapy plus ICB is not impactful in PDA (12, 40). Concomitantly with this behavior, in primary tumors, total myeloid cells, F4/80^+^ macrophages, CD206^+^ immunosuppressive TAMs, MDSCs, which are robustly immunosuppressive in PDA (18), and neutrophils were all significantly decreased, while numbers of CD8^+^ T cells were concurrently significantly increased (Figure 5, C–F, and Figure 6, A and B). We note that the observed decrease in neutrophils in autochthonous disease is in contrast to findings observed with grafted tumor systems using a distinct CCR2 inhibitor (41). Interestingly, Gem+ICB+CCR2i combination treatment also increased the frequency of iNOS^+^ cells, a marker of classically activated macrophages, in the tumor microenvironment (TME) (Supplemental Figure 6B). Along with these shifts in the immune landscape, we also identified decreased tumor cell proliferation as evidenced by reduced Ki67^+^ staining and enhanced apoptosis in the Gem+ICB+CCR2i compared with the Gem and Gem+ICB groups (Figure 6, C and D). Combination therapy also resulted in increased vascular patency (Supplemental Figure 6C) without decreasing general fibrosis (as discerned from levels of α-SMA^+^ CAFs and fibrillar collagen in the PDA stroma; Supplemental Figure 6, D and E), demonstrating that reducing TAMs derived from bone marrow not only renders PDA susceptible to ICB, but can also surmount aspects of the vascular collapse phenotype that play a key role in driving drug-free sanctuaries in PDA (42).

### Combination of CCR2 inhibition and checkpoint blockade decreases metastasis in KPC mice.

**Figure 7:**
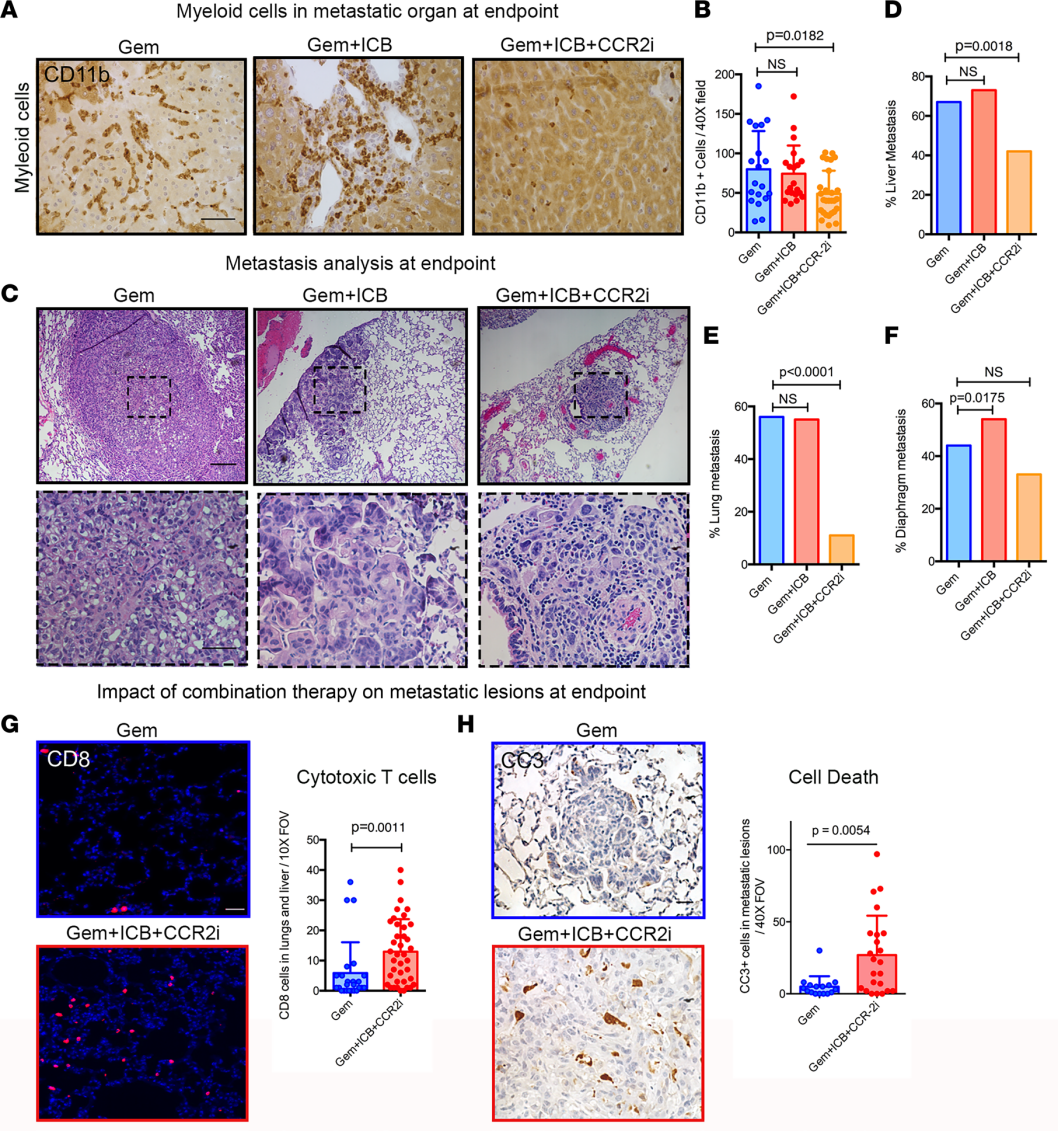
Blocking infiltration of bone marrow–derived TAMs decreases metastatic burden and increases antitumor immune responses in metastasis lesions. (**A** and **B**) IHC staining for CD11b shows that Gem+ICB+CCR2i therapy decreases myeloid cell recruitment into metastatic liver sites. *P* value by Kruskal-Wallis and Dunn’s multiple-comparison tests; *n* = 4–6 animals in each group. (**C**–**F**) Gem+ICB+CCR2i therapy significantly decreases metastatic burden. Representative images of lung metastatic lesions in Gem, Gem+ICB, and Gem+ICB+CCR2i animals (**C**) and associated quantification of metastatic burden in the liver (**D**), lung (**E**), and diaphragm (**F**) in *KPC* mice treated with Gem (*n* = 9), Gem+ICB (*n* = 11), or Gem+ICB+CCR2i (*n* = 13). (**G**) IF staining shows that Gem+ICB+CCR2i combination increases CD8^+^ T cell infiltration at metastatic sites. *P* value by Mann-Whitney test; *n* = 4–5 animals. (**H**) CC3 IHC analysis shows that Gem+ICB+CCR2i therapy increases cell death in metastatic PDA. *P* value by Mann-Whitney test; *n* = 4–5 animals in each group. Scale bars: 50 μm.

Consistent with decreased myeloid cells in primary tumors, CCR2 inhibition also blocked the accumulation of myeloid cells in metastatic organ sites (Figure 7, A and B). Therefore, to specifically determine the impact of combination Gem+ICB+CCR2i therapy on metastatic lesions, we performed a detailed necropsy and histopathological analysis of each of the 3 preclinical treatment cohorts. Analysis demonstrated that Gem+ICB+CCR2i combination therapy but not Gem or the Gem+ICB combination decreased total metastasis (Figure 7, C–F, and Supplemental Table 2). Forty-four percent of Gem+ICB+CCR2i had liver metastases compared with 66% in the Gem and 70% in the Gem+ICB cohorts (Figure 7D). Gem+ICB+CCR2i–treated mice also exhibited a profoundly lower percentage of lung metastasis (from 55 % to 11%; Figure 7E). This profound decrease in lung metastasis suggests a stronger role for marrow-derived macrophages in lung metastases. Notably, we also observed a concerning trend of increased diaphragm metastasis in the Gem+ICB, but a decreasing trend in the Gem+ICB+CCR2i cohort (Figure 7F). However, in the Gem+ICB+CCR2i cohort we again observed that decreasing myeloid cells led to a significant increase in CD8^+^ T cell population (Figure 7G) and tumor cell death (Figure 7H). We note the larger increases in cytotoxic T cell levels in metastatic sites compared with increases in primary tumors, suggesting that myeloid-targeting therapy has robust benefit for combating metastatic disease. Thus, overall these data show that, similarly to the primary disease, combination therapy can render metastatic disease susceptible to ICB, increase antitumor immunity, and increase cell death in established metastatic disease, resulting in an overall decrease in metastatic burden.

### TNF-α/IL-33 signaling resulting in increased CD8^+^ T cell infiltration.

**Figure 8:**
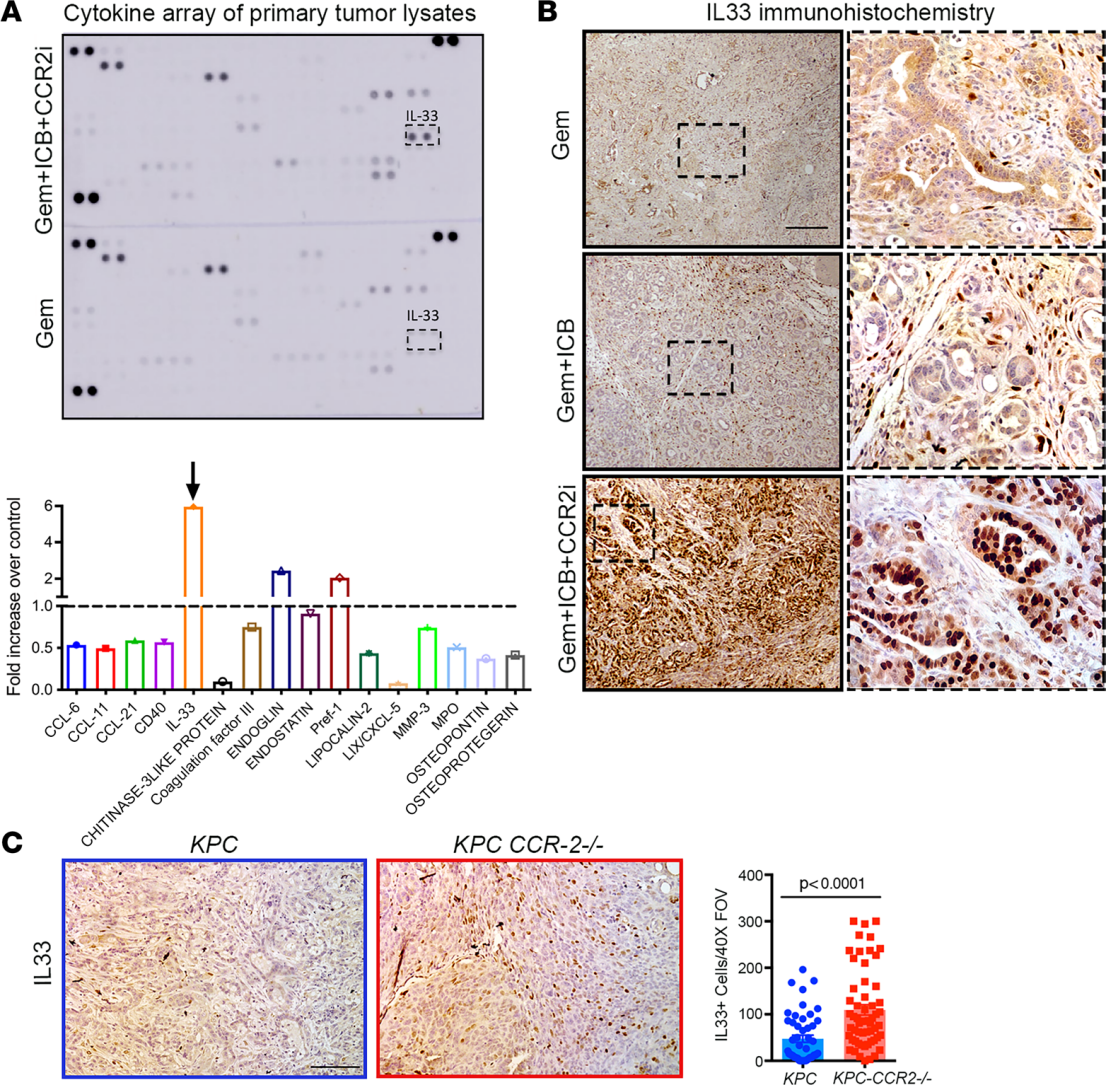
Blocking TNF-α–producing TAMs increases alarmin IL-33 levels in the TME. (**A**) Protein cytokine array of tumor lysates and quantification of array spots showing increased IL-33 expression upon Gem+ICB+CCR2i treatment (*n* = 2 tumors in each group). (**B**) IHC of IL-33 in Gem-treated, Gem+ICB–treated, and Gem+ICB+CCR2i–treated tumors, showing robustly elevated levels following Gem+ICB+CCR2i treatment. (**C**) IHC and quantification show higher IL-33 levels in *KPC*-*CCR2^–/–^* animals compared with *KPC*. *P* value by Mann-Whitney test; *n* = 4–5 animals. Scale bars: 50 μm.

To identify the signal that led to increased antitumor immunity, we measured cytokine levels in treated tumors. Among all cytokines, IL-33 was the most highly enriched in the Gem+ICB+CCR2i–treated tumors (Figure 8A). We confirmed this result in all 3 treatment groups through IHC analysis that shows a heterogeneous expression in both the carcinoma and stromal compartments in both the GEM and GEM+ICB groups with higher levels in the stroma (Figure 8B). However, in the triple therapy group we again observed expression in both compartments, but a profound increase in IL-33 expression in carcinoma cells (Figure 8B). Increased levels of IL-33 were also observed in *KPC-CCR2^–/–^* animals (Figure 8C). These data suggest that blockade of bone marrow–derived macrophages in concert with chemotherapy and immune therapy leads to robust increases in IL-33 expression in pancreatic tumors.

**Figure 9:**
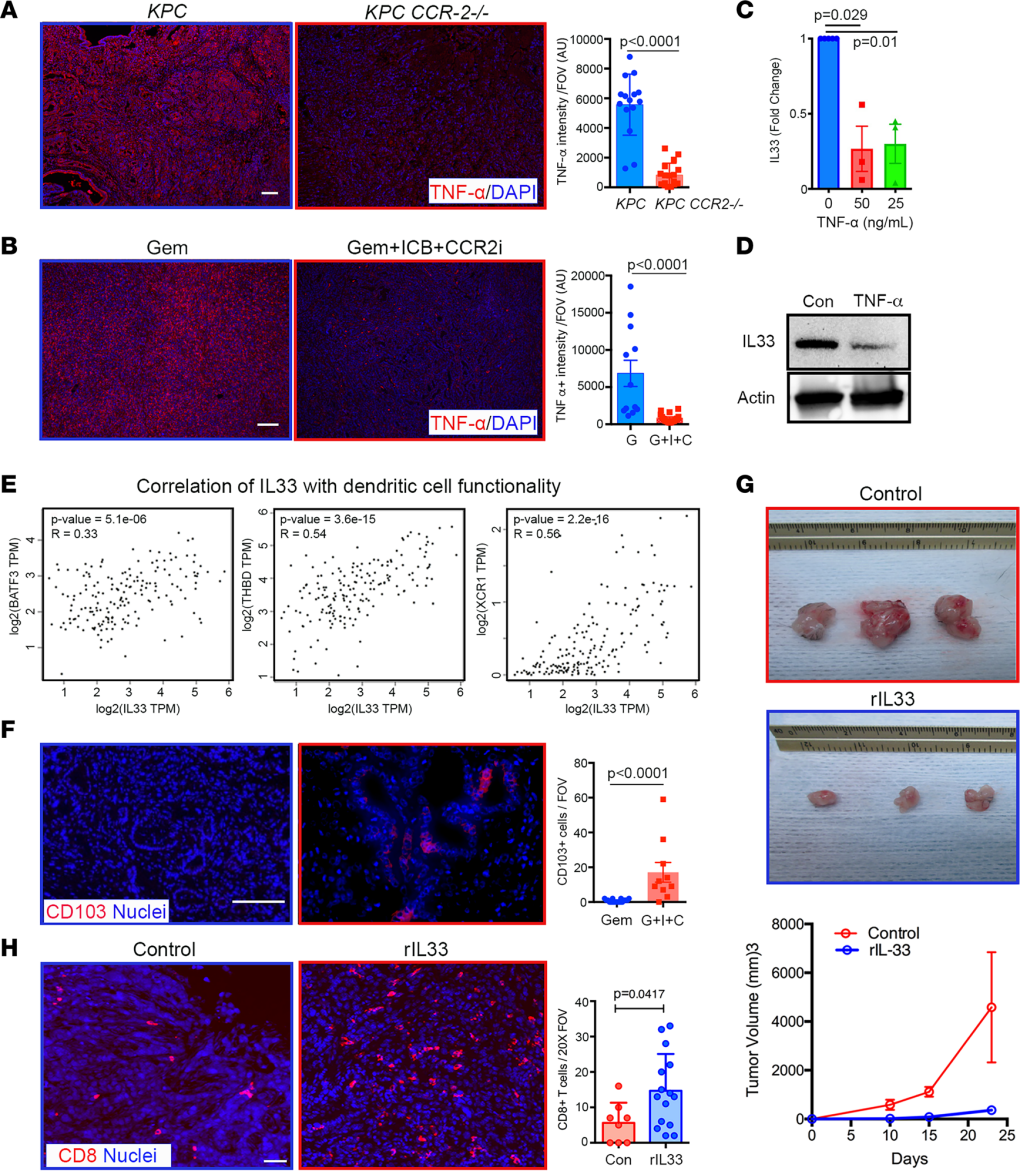
Increases in IL-33 induce increases in CD8^+^ cytotoxic T cell levels. (**A** and **B**) IF staining shows significantly decreased TNF-α levels in *KPC-CCR2^–/–^* and Gem+ICB+CCR2i treatment groups compared with *KPC* and Gem groups. *P* value by Mann-Whitney test; *n* = 4–5 animals. (**C** and **D**) Treatment of *KPC* cells with recombinant TNF-α causes a decrease in IL-33 at the gene and protein levels. *P* value by Kruskal-Wallis and Dunn’s multiple-comparison tests; *n* = 3. (**E**) Strong correlations between IL-33 and markers of CD103^+^ DC levels and functionality (TCGA data set analysis). (**F**) IF staining shows a significant increase in CD103^+^ DCs in Gem+ICB+CCR2i treatment group. *P* value by Mann-Whitney test; *n* = 4–5 animals. (**G**) Analysis of tumor growth curves for control and rIL-33 mice shows that IL-33 decreases the size of subcutaneous PDA tumors and overall tumor volume at the endpoint (*n* = 3 per group). (**H**) IF staining and quantification demonstrate that IL-33 increases CD8^+^ T cell numbers in PDA tumors. *P* value by Mann-Whitney test; *n* = 3 animals per group. Scale bars: 10 μm.

Macrophages also showed very high enrichment of TNF-α production pathways (Figure 1E). Indeed, dual staining of *KPC* tumor showed that most of the TNF-α colocalized with F4/80^+^ macrophages (Supplemental Figure 7A), and analysis of RNA-Seq data showed strong expression from TAMs (Supplemental Figure 7B), validating the conclusion that macrophages are a primary source of TNF-α in PDA, consistent with previous findings (43, 44). Further, immunofluorescence (IF) analysis of both tumors from *KPC-CCR2^–/–^* mice and tumors treated with combination therapy showed a profound decrease in TNF-α levels (Figure 9, A and B), again supporting the conclusion that tumor-infiltrating bone marrow–derived TAMs are a primary source of TNF-α in PDA. Previously, TNF-α has been shown to modulate the expression of IL-33 in normal and diseased fibroblasts (45). Thus, next we hypothesized that this increase in IL-33 could be due to a decrease in TNF-α from macrophages. Since the major increase in IL-33 in the Gem+ICB+CCR2i group was observed from carcinoma cells, we sought to test whether TNF-α can directly regulate IL-33 expression in carcinoma cells. We treated primary *KPC* cell lines with recombinant TNF-α, which led to significantly decreased IL-33 at both the gene and protein levels (Figure 9, C and D). Interestingly, IL-33 is a member of the IL-1 family that has been shown to play a key role in innate and adaptive immunity (46, 47). IL-33 is normally released by damaged or necrotic cells and can act as an alarmin capable of activating either Th1 or Th2 response (47, 48). Furthermore, IL-33 was recently shown to activate tumor-infiltrated group 2 innate lymphoid cells (ILC2s) to regulate CD8^+^ T cell responses (49). Indeed, IL-33 expression correlated strongly with CD8A and granzyme B as well as genes like *BATF3*, *IRF8*, *THBD*, *CLEC9*, and *XCR1* that are required for tumor antigen cross-presentation functionality of CD103^+^ dendritic cells (DCs) (Figure 9E and Supplemental Figure 7C). Therefore, to test whether decreased TNF-α and increased IL-33 do indeed lead to increased numbers of CD103^+^ DCs in the tumor microenvironment, we stained the tumor sections for both CD11c and CD103 and observed a significant increase in both DC markers (Figure 9F and Supplemental Figure 7D). Next, we subcutaneously implanted *KPC* cells in syngeneic mice with or without recombinant IL-33 (rIL-33) mixed into growth factor–reduced Matrigel. Consistent with evidence suggesting that increased IL-33 levels promote antitumor T cell responses, PDA tumor growth was profoundly inhibited in the rIL-33 group compared with control conditions (Figure 9G) with concomitant and robust increases in CD8^+^ T cell infiltration (Figure 9H). We note that, consistent with findings by Moral et al. (49), we did not observe IL-33R expression of CD8^+^ T cells (Supplemental Figure 7, E–G), suggesting that IL-33 signaling impacts T cells through an intermediary, such as ILC2s and CD103^+^ DCs. These data therefore show that the alarmin IL-33 promotes CD8^+^ T cell infiltration and antitumor response in PDA. Thus, we demonstrate that depletion of immunosuppressive TAMs and MDSCs results in decreased TNF-α, causing increased IL-33 levels in carcinoma cells that result in increased cytotoxic T cell response to combat metastatic PDA.

## Discussion

Recent studies across a range of cancer have greatly advanced our understanding of the suppression of host immunity by TAMs (22, 50–52). However, macrophage diversity, complexity, and regulatory mechanisms in many tumors, including those of the pancreas, remain poorly defined. Our findings highlight the interpatient and intratumor TAM heterogeneity present in patients with PDA and its faithful recapitulation in preclinical murine models. While additional studies are needed to parse out and define the impact of this heterogeneity, it likely contributes to the patient-specific tumor biology and therapeutic responses. Indeed, while analysis shows that most PDA TAMs display a more M2-like polarization and high immunosuppressive capacity, substantial variability exists within and across tumors, suggesting that a better understanding of both spatial and temporal TAM dynamics is needed. However, we do note that most of the immunosuppressive and protumor TAM genes overlapped with markers suggestive of a bone marrow origin and that limiting large portions of this heterogeneous population by disrupting monocyte homing to PDA is therapeutically beneficial. In fact, blocking infiltration of bone marrow–derived TAMs significantly relieves immune suppression and augments checkpoint blockade therapy. Previously, in grafted tumor models, targeting CCR2 resulted in a compensatory influx of neutrophils (41), something we did not observe here using a different CCR2 inhibitor against autochthonous disease. Furthermore, we observed strong TNF-α signaling from bone marrow–derived TAMs. Interestingly, TNF-α is an abundant cytokine in PDA TME (53), yet the role of TNF-α in tumor development remains paradoxical and has been shown to be antitumor and protumor in different contexts. For instance, treatment of PDA cells with recombinant TNF-α increased EGFR expression from carcinoma cells (54), while in another study recombinant TNF-α promoted the growth of Panc02 tumors in mice but inhibited *KPC* cell growth (55). Furthermore, a recent study suggests that higher expression of TNF-α forces classical neoplastic cells into an aggressive basal-like state suggesting a protumorigenic role for TNF-α in PDA (43). Then there are additional roles in regulating tumor stromal dynamics and immunity. Our study suggests that TNF-α directly regulates the expression of IL-33 in carcinoma cells, thus supporting the conclusion that TNF-α plays a role in immune suppression in PDA that can be overcome through blockade of bone marrow–derived macrophages in concert with immune therapy. Thus, targeting broad and diverse collections of protumor immunosuppressive TNF-α–producing TAMs appears to be part of a viable strategy to break down one of the key barriers to effective antitumor immunity in PDA.

In recent years preclinical and clinical studies have sought to disrupt myeloid cell levels and/or function to improve outcomes. For instance, inhibiting CSF1R signaling functionally reprograms macrophage responses to enhance antigen presentation and productive antitumor T cell responses in orthotopic grafted tumor PDA models (56). Unfortunately, the CSF1R-blocking monoclonal antibody cabiralizumab combined with nivolumab and chemotherapy in advanced PDA did not improve progression-free survival in a phase II clinical trial (ClinicalTrials.gov NCT03336216). Further, in another recent study, CSF1R inhibition by PLX5622 caused long-term changes in bone marrow–derived macrophages and also reduced the population of resident and interstitial macrophages of peritoneum, lung, and liver, raising concerns about the potential long-term consequences of CSF1 inhibition (57). Yet, targeting MDSCs and TAMs remains a viable strategy to overcome one of the major obstacles to effective antitumor immune responses in PDA. For instance, targeted depletion of granulocytic MDSCs in autochthonous PDA in *KPC* mice increases the intratumoral accumulation of activated CD8^+^ T cells (16), while partial activation of CD11b leads to TAM repolarization, a reduction in immunosuppressive myeloid cells, and enhanced DC responses to improve antitumor T cell immunity (14). Moreover, targeting CCR2 has also emerged as a strategy to improve outcomes since it has been hypothesized to reduce infiltration of myeloid cells. Indeed, in a grafted tumor model, CCR2 inhibition was effective at reducing TAMs (20, 34), which is consistent with our finding here in autochthonous primary and metastatic disease that TAM reductions render PDA susceptible to ICB. In 2 different clinical trials, CCR2 antagonists have been combined with chemotherapy (58, 59) in an attempt to overcome chemoresistance in pancreatic cancer patients, yet to our knowledge, CCR2 inhibition in concert with immune therapy has not yet been trialed in PDA. However, the data from these trials suggest that CCR2 inhibitors are well tolerated in patients and thus could likely be safely combined with immune checkpoint inhibitors to overcome immune resistance. As such, novel ways to target myeloid cell infiltration, such as the new CCR2 inhibitor used here, or through manipulation of key signaling pathways such as IL-33 signaling, appear to be viable strategies for improving outcomes in PDA. Likewise, we note that our analysis demonstrates that both myCAFs and iCAFs produce significant amounts of CCL2 (Figure 3 and Supplemental Figure 3), suggesting that targeting CAFs also has the potential to improve antitumor immunity, consistent with reports of targeting of CXCR4 or FAK signaling to increase susceptibility to ICB (12, 13). However, care must be taken to overcome barriers of CAF signaling, as manipulation of the stroma can also lead to more aggressive disease in some contexts, particularly when undertaken prior to the onset of disease, but can be very beneficial in other cases, including increased influx of CD8^+^ T cells and M1 macrophages after antifibrotic therapy (15), highlighting the complex role of the TME in disease pathogenesis and therapeutic response. Yet it is clear that we must find ways to overcome these barriers to effective distribution of therapies and antitumor immunity. Indeed, it appears likely that combinations of stroma-targeting therapies (targeting CAFs, ECM, etc.) and myeloid suppression, perhaps with immune priming, in concert with molecular or cellular immune therapies will be needed to overcome patient-specific stromal and immunosuppressive barriers in PDA to improve patient outcomes.

Last, our data also suggest that blocking bone marrow–derived TAMs leads to a profound decrease in TNF-α and increased IL-33 in PDA. IL-33 is a Th1 and Th2 promoter cytokine and an alarmin that can be released from cells during cell death (60, 61). IL-33 has also been suggested to have both protumor (62, 63) and antitumor (49, 64, 65) roles. In our study, increased IL-33 due to decreased immunosuppressive myeloid cells/TNF-α resulted in increased CD103^+^ DCs and CD8^+^ cytotoxic T cells, particularly in metastatic sites, supporting the previous finding that PDA patients with higher IL-33 survive longer and that IL-33 can promote antitumor immune responses by actively ILC2s that can indirectly prime CD8^+^ T cells, likely through recruitment of CD103^+^ DCs that promote T cell recruitment and priming (49). In the same study, intraperitoneal injection of rIL-33 activated ILC2s in orthotopic tumors, but this was not the case for a subcutaneous grafted murine tumor model. However, in our study, a direct implant of rIL-33 in the developing tumor, which we believe well represents elevated IL-33 levels in the tumor microenvironment, led to increased T cell response and decreased tumor growth suggestive of a global role of IL-33 in activating antitumor immunity. Thus, put all together, our study suggests a novel therapeutic strategy to decrease TNF-α and/or increase IL-33 in PDA patients that could lead to better outcomes. Indeed, these collective data suggest a potential for multiple therapeutic avenues, including targeting of TAMs and MDSCs, anti–TNF-α therapy, direct manipulation of IL-33 levels, or alteration of the behavior of ILC2s and DCs. Thus, in summary, our study reports heterogeneous TAM populations that originate from bone marrow that are the primary source of TNF-α. *Ccr2* deficiency decreases bone marrow–derived TAMs and thus TNF-α, leading to increases in IL-33 and thus increasing survival and decreasing metastasis in *KPC* mice. Furthermore, blocking of marrow-derived macrophages in combination with gemcitabine and ICB increases survival and decreases metastasis, suggesting a rational combination strategy to activate antitumor immunity in PDA patients.

## Methods

### Single-cell data analysis.

Human single-cell data (phs001840.v1.p) processed by Cell Ranger version 1.3.1 (10x Genomics) (27) were loaded to the R package 2.3.1. Cell clusters were identified via the FindClusters function using a resolution of 0.6 for all samples, based on a graph-based clustering algorithm. Functional enrichment of Gene Ontology (GO) and KEGG pathway analyses were performed using ToppGene (https://toppgene.cchmc.org). *P* values less than 0.05 were considered significant enrichment. Mouse single-cell data (GSE125588) processed by Cell Ranger version 1.3.1 (10x Genomics) (32) were loaded to the R package Seurat version 2.3.1. Cell clusters were identified via the FindClusters function using a resolution of 0.6 for all samples, based on a graph-based clustering algorithm. A likelihood ratio–based test or an AUC-based scoring algorithm was used to compute marker genes for each cluster, and expression levels of several known marker genes were examined. Different clusters expressing known marker genes for a given cell type were selected and combined as 1 for each cell type. Macrophage subclusters were then further identified in macrophage clusters using the SetAllIdent function.

### The Cancer Genome Atlas human patient cohort data analysis.

Transcript analysis was performed on human data sets publicly available through The Cancer Genome Atlas database. Correlation analysis was performed using the GEPIA platform (66).

### Generation of murine primary KPC carcinoma cells, iCAFs, and myCAFs.

All murine primary cell lines were generated from freshly excised *KPC* tumors as described previously (15). iCAFs were generated according to a previously reported protocol (36). In brief, for iCAFs, the purified pancreatic stellate cells were plated in Matrigel and were cultured in tumor organoid condition media for 3 days, and for myCAFs, the purified CAFs were plated directly on a 2D surface. The phenotype of iCAFs and myCAFs was confirmed by measurement of the gene expression of *Il-6*, *Cxcl1*, *Acta-2*, *Lif*, and *Ctgf* (Supplemental Figure 3C).

### TNF-α treatment.

*KPC* cells were grown on minimal media (DMEM with 1% FBS) overnight, and then cells were treated with different concentrations of recombinant TNF-α for 24 hours. After 24 hours the cells were harvested for protein and RNA isolation. Western blot was done as described previously (67).

### Real-time PCR analysis.

Total RNA was extracted with Trizol (Roche) as described by the manufacturer, and for cDNA synthesis, 1 μg of total RNA was reverse-transcribed using the SuperScript II (Thermo Fisher Scientific) Reverse Transcription System. Real-time PCR was run in triplicate using Platinum SYBR Green master mix (Bio-Rad Laboratories), following the manufacturer’s instructions. Real-time monitoring of PCR amplification was performed using the qPCR System (Roche). Data were expressed as relative mRNA levels normalized to 18S, which served as endogenous normalization control expression level in each sample, and are represented as mean ± SEM between 3 independent experiments unless otherwise indicated in the figure legend. The primer sequences are listed in Supplemental Table 3. Pre-made *Ccl2* primers were obtained from Sino Biological (MP200388) and 18S from Qiagen (catalog 249900).

### Animal studies.

*Ccr2^–/–^* mice (strain 004999) were obtained from The Jackson Laboratory. To generate *KPC-CCR2^–/–^* mice, *Kras^LSL-G12D^* *Pdx1-Cre* (*KC*) mice were crossed with *p53^LSL-R172H/+^* or *p53^LSL-R172H/LSL-R172H^* mice to generate *KPC* mice. We crossed *CCR2^–/–^* mice with *KC* mice to generate *KC-CCR2^–/–^* mice, and these were then crossed with *p53^R172H/+^* or *p53^LSL-R172H/LSL-R172H^* mice to generate *KPC-CCR2^–/–^* mice. The *KPC-CCR2^–/–^* mice were of C57BL/6 background. *KPC* mice on the C57BL/6 background were used as the control for the comparative studies. The progeny were born in an expected Mendelian ration, with no obvious functional defects. In vivo drug studies were done using a *KPC* genetically engineered mouse model (68) on a mixed background as previously described. At the endpoint (or, for the early studies, at 10–11 weeks), full necropsies were performed on all study animals and included a gross examination of all organs for macroscopic disease as previously described (15, 67, 69).

### Histological analysis.

All stained tissue samples were digitally scanned at high resolution and viewed using a Nikon Ti-3 microscope. Using this software, we identified the PDA-positive area within H&E-stained sections. PanINs, PDA, and necrotic and metastatic lesions were identified on H&E and were quantified according to previously reported protocols (67). For histopathological analysis, high- and low-grade PanINs or PDA and high- and low-grade tumors were quantified as a percentage relative to the total tumor. Invasion of the tumor cells into the surrounding stroma was histologically scored on a scale of 0 to 2, with 0 indicating no invasion and 2 indicating high invasion, and invasion was defined as tumor cells penetrating the adjacent organs such as the duodenum, liver, etc.

### Flow cytometry.

To quantitate blood monocytes, 200 μL of blood was obtained by submandibular vein, incubated in red blood cell lysis buffer (BioLegend) for 15 minutes on ice, and stained with fluorophore-conjugated antibodies for 20 minutes on ice. For ST2 receptor detection, total spleen cells were isolated from C57BL/6 mice in ice-cold PBS and stained with ST2 antibodies for 15 minutes on ice. Stained cells were analyzed on an LSR-II flow cytometer (BD Biosciences). Antibodies utilized for flow cytometry, and other staining or Western blotting as noted below, are described in Supplemental Table 4.

### ELISA.

CCL2 levels were quantified in cell culture media using commercially available ELISA kits (Thermo Fisher Scientific, 88-7391-22) according to the manufacturer’s instructions. Briefly, cells were seeded into 24-well plates. After 24 hours the culture supernatant was collected and centrifuged (5,000*g* for 5 minutes), and secreted CCL2 was measured by the ELISA kit.

### Western blot.

Cells were collected in ice-cold RIPA buffer supplemented with a complete protease inhibitor cocktail (Roche, 11697498001). Protein concentration was determined by Bradford assay. Equal amounts of proteins were resolved via 4%–20% (vol/vol) SDS-PAGE and electrophoretically transferred onto nitrocellulose membranes using a semi-dry blotting system (Bio-Rad, 10026938). Membranes were blocked with 5% (wt/vol) milk powder in TBST for 1 hour at room temperature, followed by incubation with primary antibodies overnight at 4°C. Secondary HRP-linked antibodies were incubated for 1 hour at room temperature. Protein was detected by a Bio-Rad Imager using ECL substrate (Millipore). β-Actin was used as a loading control.

### Ultrasound for enrollment and monitoring of disease progression.

Weekly ultrasound was performed on *KPC* mice using the Vevo 2100 Imaging System for both enrollment and disease monitoring. When tumors reached a diameter of 4–8 mm, mice were randomly enrolled in study groups. The Vevo 2100 software was used to reconstruct 3D tumor volumes for quantification of tumor volume growth over time.

### In vivo drug treatment experiments.

For drug treatments, mice with 4 to 8 mm tumors in the longest direction were randomly assigned to cohorts. The treatments used were gemcitabine (LKT Laboratories, 61745) at 100 mg/kg by i.p. injection every 4–5 days, CCR2 inhibitor (CCX598 compound, provided by ChemoCentryx LLC) at 100 mg/kg per os (70) daily, and anti–PD-1 and anti–CTLA-4 (Bio X Cell) injection i.p. every 4–5 days at 250 and 200 μg, respectively.

### IHC staining.

Formalin-fixed, paraffin-embedded (FFPE) tissue sections (5 μm) were stained for the targets according to a previously reported protocol (15, 69, 71). In brief, for α-SMA and CD31, antigen retrieval was performed using 1× citrate buffer, pH 6 (Sigma), for 20 minutes, washing with 1× TBS/0.1% Tween-20 (TBST; all washes were done in TBST), blocking with 5% goat serum for 1 hour, then incubation with primary antibody overnight at 4°C. This was followed by an endogenous peroxidase block with 3% H_2_O_2_ for 15 minutes, incubation with a polymer secondary antibody for 30 minutes (rat probe, BioCare Rat-on-Mouse anti-CD31 Polymer Kit) followed by rat-HRP (BioCare) for 30 minutes, and then counterstaining with freshly filtered Mayer’s hematoxylin (Fisher Scientific) for 5 minutes followed by dehydration and clearing with increasing concentrations of ethanol and xylenes. For α-SMA, antigen retrieval was performed using a 1× Dako Tris/EDTA, pH 9, solution for 20 minutes. This was followed by an endogenous peroxidase block of 3% H_2_O_2_ for 10 minutes, blocking for avidin/biotin (Vector kit), blocking with TCT buffer (0.1% trypsin, 0.1% CaCl_2_, 20 mmol/L Tris-Cl pH 7.8) for 10 minutes, then incubation with primary antibody for 1 hour at room temperature. Slides were then incubated in MACH2 rabbit–HRP, followed by incubation with DAB for 10 minutes, counterstaining with hematoxylin, and finally dehydration and clearing. For iNOS, IL-33, S1009A, F4/80, CD206, CCL2, FN1, CD11c, and cleaved caspase-3, antigen retrieval was performed using 1× citrate buffer, pH 6 (Sigma), for 30 minutes and washing with 1× TBS/0.1% Tween-20 (TBST) followed by an endogenous peroxidase block by 3% H_2_O_2_ for 15 minutes and then blocking with 5% goat serum for 1 hour, then incubation with primary antibody overnight at 4°C. This was followed by incubation with anti-rabbit– or anti-rat–HRP polymer secondary antibody for 30 minutes (BioCare Polymer Kit), followed by incubation with DAB, and then counterstained with freshly filtered Mayer’s hematoxylin. Details for the antibodies used are provided in Supplemental Table 4.

### Immunofluorescent staining.

OCT compound–embedded frozen tissue sections were stained for Ly6G, CD103, and anti–pan-cytokeratin. Slides were air-dried for 15 minutes at room temperature, incubated in acetone for 15 minutes, and then air-dried for 15 minutes. Slides were rehydrated with 1× PBS for 10 minutes, blocked with 2% normal goat serum for 1 hour at room temperature, and incubated with primary antibody for 2 hours at room temperature. Then, secondary antibody (1:1,000; Life Technologies, 1749750) and directly conjugated antibodies (pan-cytokeratin) were added for 1 hour at room temperature, followed by counterstaining with Bisbenzimide (1:10,000; Sigma-Aldrich) for 10 minutes at room temperature and mounting with Prolong Gold (Life Technologies). Washes between steps were done using 1× PBS. IF samples were imaged on a Nikon Ti-U fluorescence microscope. For staining in FFPE samples, CD8 primary antibody was detected with a Tyramide SuperBoost kit (Invitrogen, B40944) according to the manufacturer’s instructions. Heat-induced antigen retrieval was performed with Epitope Retrieval Buffer 2 (Dako pH 9) for 20 minutes. Primary antibodies were incubated overnight at 4°C. The nonspecific background was blocked with 3% normal donkey serum plus 3% normal goat serum in PBS-T. To quantify IHC- or IF-stained tissue sections, 10–12 regions of interest were randomly selected within the PDA-positive area, defined by referencing of previously identified PDA-positive areas in adjacent H&E-stained sections. The numbers of positive cells were calculated using ImageJ (NIH). TNF-α antigen retrieval was done in citrate buffer for 20 minutes, and slides were incubated overnight at 4°C. The primary antibody was detected by goat anti-mouse IgG (Thermo Fisher Scientific, A-21124). Details for the antibodies used are provided in Supplemental Table 4.

### Collagen imaging.

Second-harmonic generation imaging was implemented on fibrillar collagen in FFPE tissue sections that were rehydrated with xylenes and serial dilutions of ethanol, then mounted with Prolong Gold (Life Technologies). Visualization of collagen was done on a custom-built multiphoton laser scanning microscope (Prairie Technologies/Bruker) using a Mai Tai Ti:Sapphire laser (SpectraPhysics) that we have previously described (72) at an excitation wavelength of 880 nm as previously described (73, 74).

### Protein cytokine assays.

Protein cytokine array was performed according to the manufacturer’s instructions (ARY028, Proteome Profiler Mouse XL Cytokine Array, R&D Systems). In brief, tissues were homogenized in PBS with protease inhibitors. After homogenization, Triton X-100 was added to a final concentration of 1%. Samples were frozen at ≤–70°C and thawed, followed by centrifugation at 10,000*g* for 5 minutes to remove cellular debris. Two hundred grams of protein was used per assay.

### Recombinant IL-33 implants.

*KPC* cells (25,000) in suspensions in Matrigel were injected s.c. into the rear right flank of C57BL/6 mice (6–8 weeks old) mixed with either rIL-33 (1 μg; R&D Systems, AF3626) or PBS. Tumor size was measured every 4–5 days. Tumor volumes were measured along orthogonal axes (*a*, *b*, and *c*) and calculated as *abc*/2.

### Statistics.

Data were tested for the assumption of normality using the Shapiro-Wilk normality test. Normally distributed 2-group data were analyzed using a 2-tailed *t* test (for 2 groups) or 1-way ANOVA (for multiple comparisons). For data that did not pass the normality test, the nonparametric Mann-Whitney sum rank test (for 2 groups) or Kruskal-Wallis (for multiple groups) followed by Dunn’s multiple-comparison test was performed. Kaplan-Meier survival data were analyzed using a log-rank test. Metastatic disease burden was analyzed using Fisher’s exact test.

### Study approval.

All animal studies were approved by the IACUC of the University of Minnesota. All human samples were deidentified and obtained either through the UMN BioNet Shared Resource program at the University of Minnesota in accordance with University of Minnesota IRB approval that includes written informed consent for tissue donation, or from publicly available commercial sources.

## Author contributions

AD and PPP conceptualized and designed the study. AD and PPP participated in the design of experiments, and AD, JZ, and HH performed experiments. AD performed mouse experiments with assistance from JZ. AD, AS, HH, KZ, RAB, and PPP participated in data analysis and interpretation, including analysis of unique data sets and quantitative metrics and algorithms. AD, PPP, and AS designed bioinformatics analysis for this study, and AS and HH performed bioinformatics analysis. AD, AS, HH, and PPP performed statistical analyses of data. PPP, AS, and RAB secured funding. AD and PPP wrote the manuscript, with significant input by RAB. All authors read and contributed comments to the final manuscript. PPP oversaw all aspects of the study.

## Supplementary Material

Supplemental data

Supplemental table 1

Supplemental table 2

Supplemental table 3

Supplemental table 4

## Figures and Tables

**Table 1:**
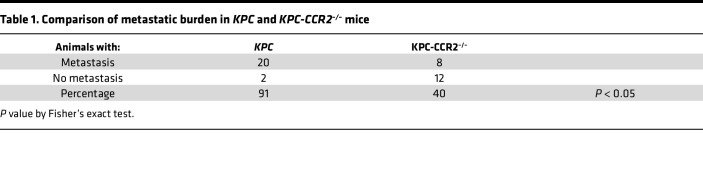
Comparison of metastatic burden in *KPC* and *KPC-CCR2^–/–^* mice

## Version 1 10/18/2022

In-Press Preview

## Version 2 11/22/2022

Electronic publication
