# Supplementary material for: Targeting TNF-α–producing macrophages activates antitumor immunity in pancreatic cancer via IL-33 signaling
Source: JCI Insight. 2022 Nov 22;7(22):e153242. doi: 10.1172/jci.insight.153242 (PMC9746819; doi:10.1172/jci.insight.153242)
Supplement: Supplemental data [file jciinsight-7-153242-s191.pdf]

Supplementary Information:

Targeting TNF-α producing macrophages activate antitumor immunity in pancreatic cancer through IL33 signaling

Ajay Dixit[1-3], Aaron Sarver[3], Jon Zettervall[1,2], Huocong Huang[4], Kexin Zheng[1],
Rolf A. Brekken[4], Paolo Provenzano[1,2,3,5,6#]

Affiliations:

[1] Department of Biomedical Engineering, University of Minnesota
[2] University of Minnesota Physical Sciences in Oncology Center
[3] Masonic Cancer Center, University of Minnesota
[4] Hamon Center for Therapeutic Oncology Research and Department of Surgery, UT Southwestern, Dallas, TX
[5] Institute for Engineering in Medicine, University of Minnesota
[6] Stem Cell Institute, University of Minnesota
To whom correspondence should be addressed:

Correspondence:
Dr. Paolo Provenzano
Department of Biomedical Engineering
University of Minnesota
7-120 NHH
312 Church St SE
Minneapolis, MN, 55455
e-mail: pprovenz@umn.edu

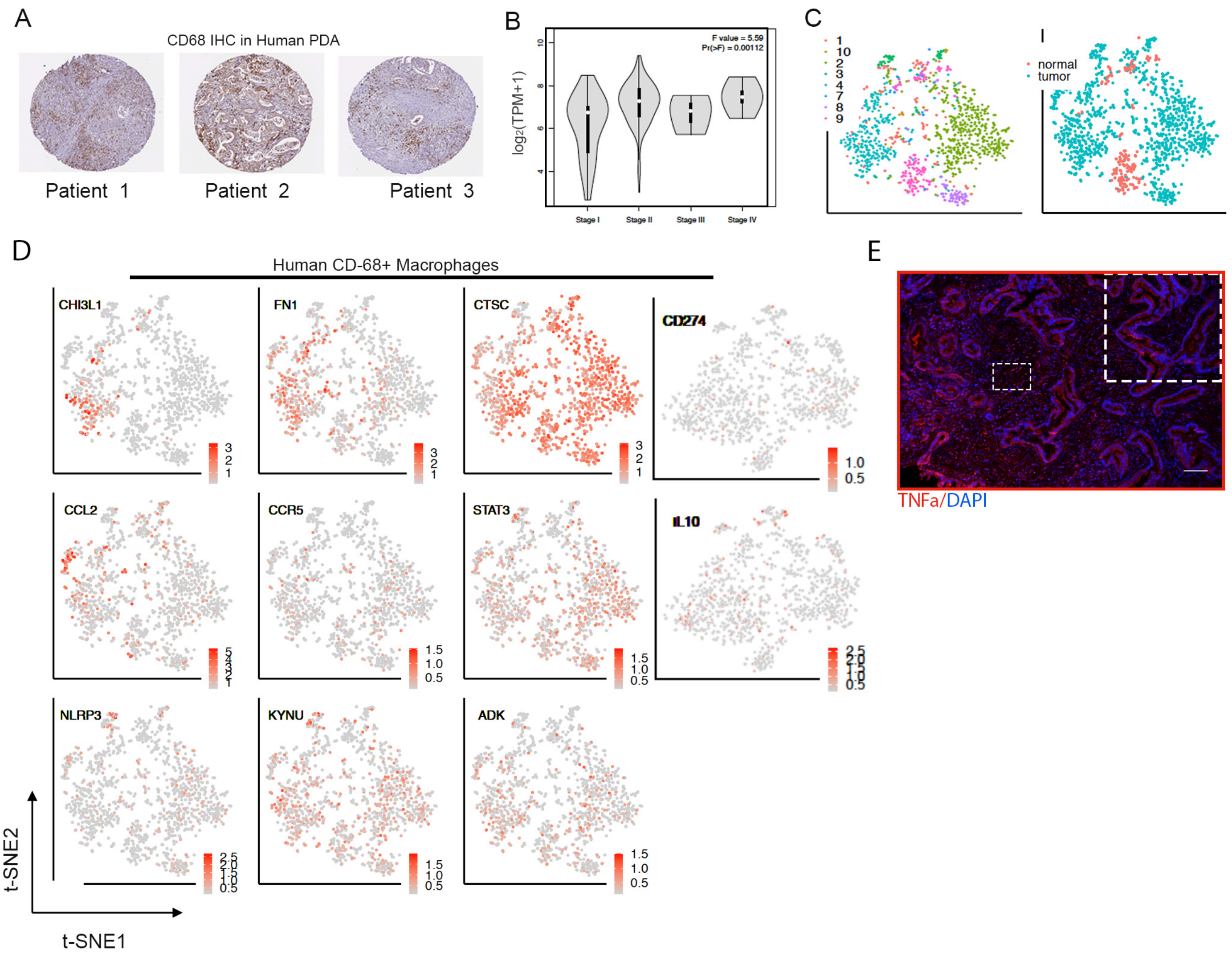

Supplementary Figure 1: Analysis of macrophages in human PDA.) IHC analysis of CD68$^+$ macrophages shows that macrophages accumulate in human PDA (TGCA data set). **B)** CD68$^+$ macrophages accumulate in early disease. Stage comparison of CD68$^+$ macrophages in human PDA shows significant transcript levels in early stage disease that continues with each stage and is highest in Stage IV. **C)** t-SNE (t-distributed stochastic neighbors embedding plot) of CD68$^+$ macrophages population in PDA showing inter- and intra-patient heterogeneity, and their sample origin. **D) D)** Visualization of different transcript distributions in human macrophage population using t-SNE (t-distributed stochastic neighbors embedding) show substantial inter- and intra-tumor heterogeneity and display pro-tumor and robust immune-suppressive pathway activation. **E)** IF staining showing TNF-α expression in infiltrating cells (Scale bar=50 μm).

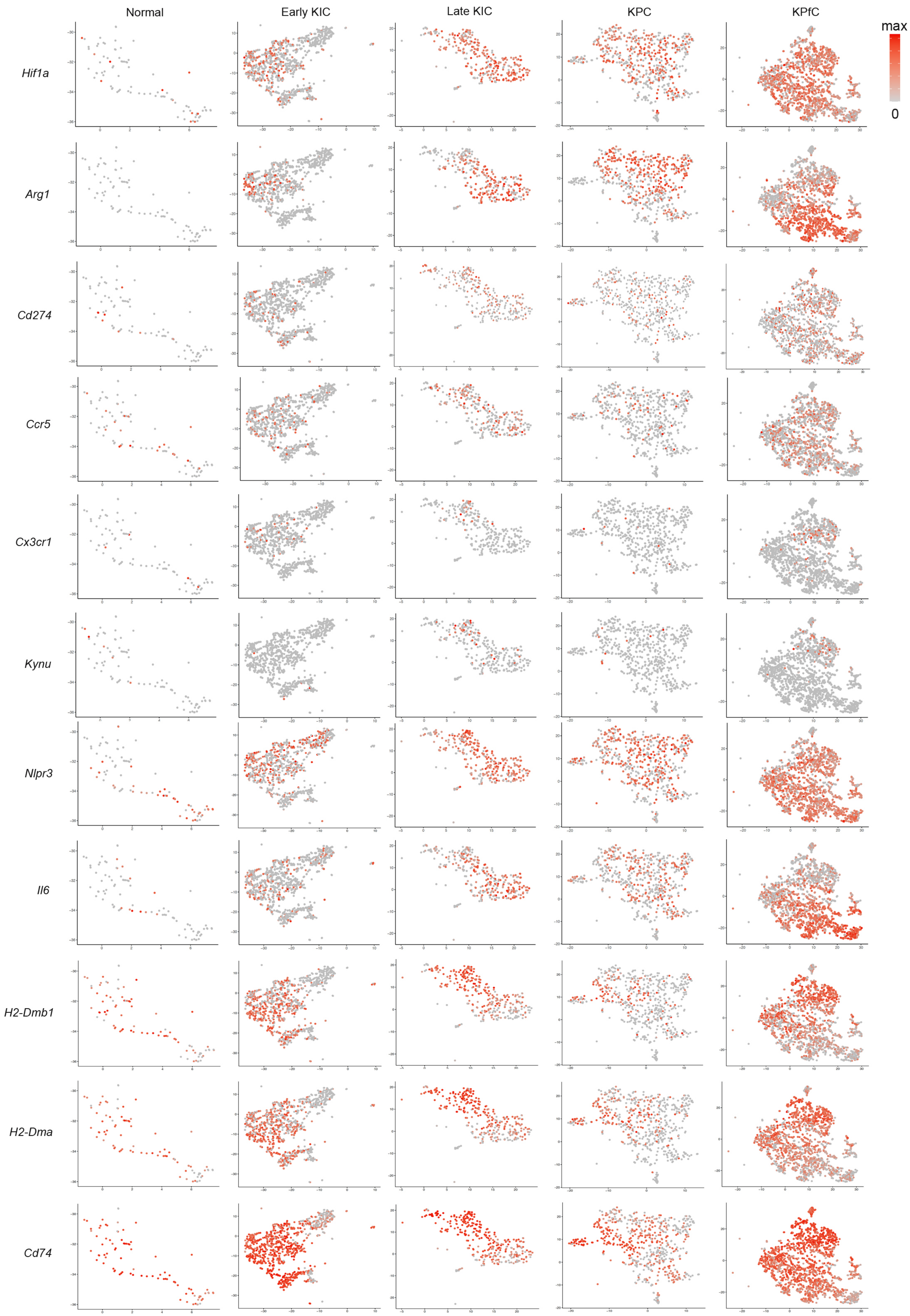

Normal
Early KIC
Late KIC
KPC
KPfC
max
0
Hif1a
Arg1
Cd274
Ccr5
Cx3cr1
Kynu
Nlpr3
Il6
H2-Dmb1
H2-Dma
Cd74

Supplementary figure 2: Cross species examination of macrophages from *KPC, KIC,* and *KPfC* genetically engineering murine models of PDA demonstrates similarities to human macrophages. Visualization of transcript distributions in murine macrophage populations using t-SNE (t-distributed stochastic neighbors embedding) show that similar to human PDA, expression patterns in *KPC, KIC*, and *KPfC* macrophages suggest robust immunosuppressive behavior.

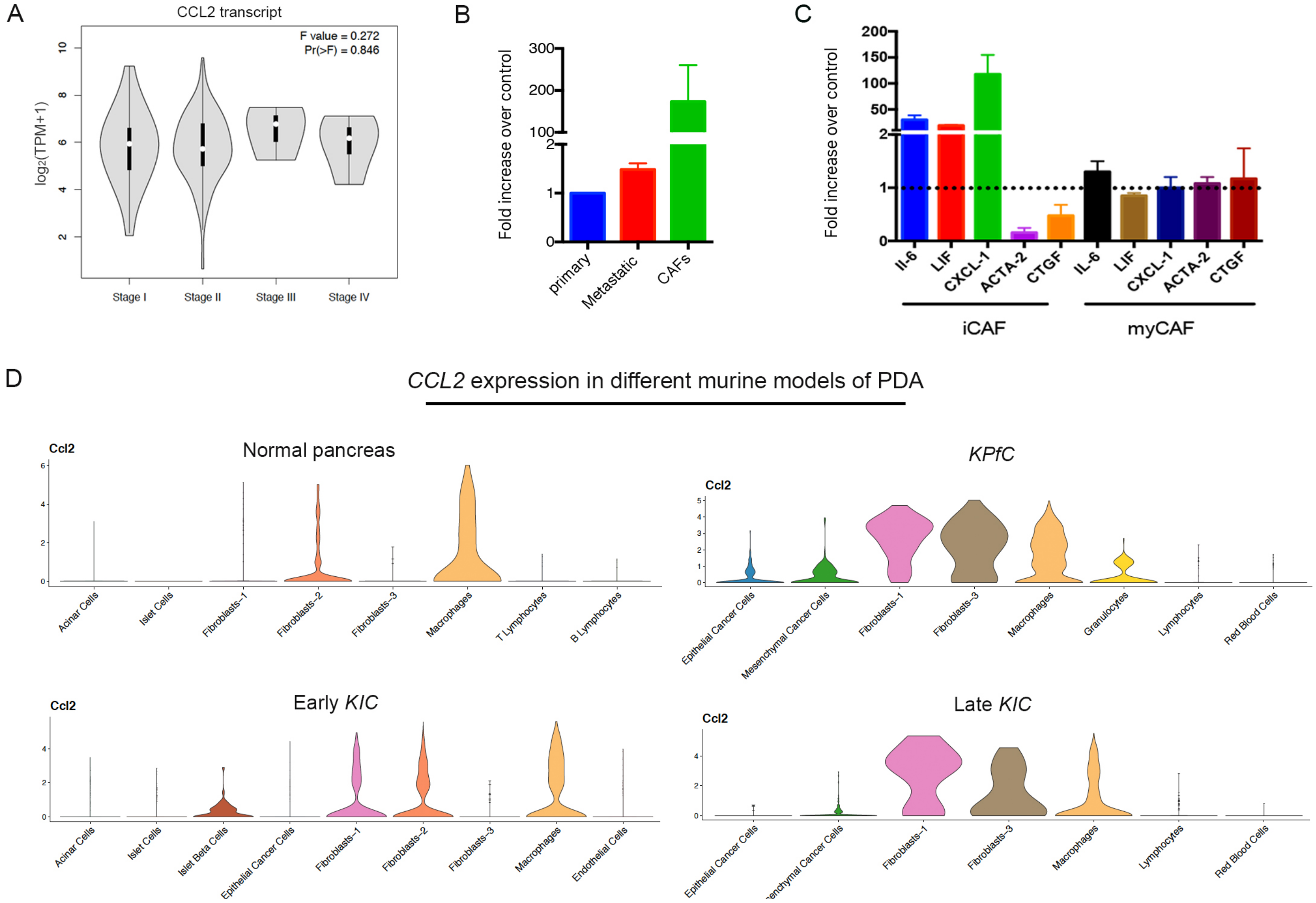

Supplementary figure 3: Stromal CAFs recruits TAMs in PDA. A) Expression of *CCL2* in stage 1-4 PDA showing *CCL2* transcript is expressed strongly in early disease and throughout all stages. **B**) Gene expression of *Ccl2* in primary and metastatic carcinoma cells and CAFs measured by qPCR showing that CAFs express greater amounts of *Ccl2* than carcinoma cells **C)** Validation of in vitro derived iCAFs and myCAFs by measuring expression of various key markers for iCAF and myCAF phenotypes. **D)** Violin plots of *Ccl2* in tumor cell populations from *KPC, KIC* and *KPfC* mouse models of PDA showing that CAFs are the major contributor of CCL2 in PDA TMEs. *KIC* mice were 60 days old (n = 3, late KIC) and KPfC (n = 1). Early *KIC* mice were sacrificed at 40 days old (n = 2), and normal pancreas obtained from mice (n = 2) sacrificed at 60 days old.

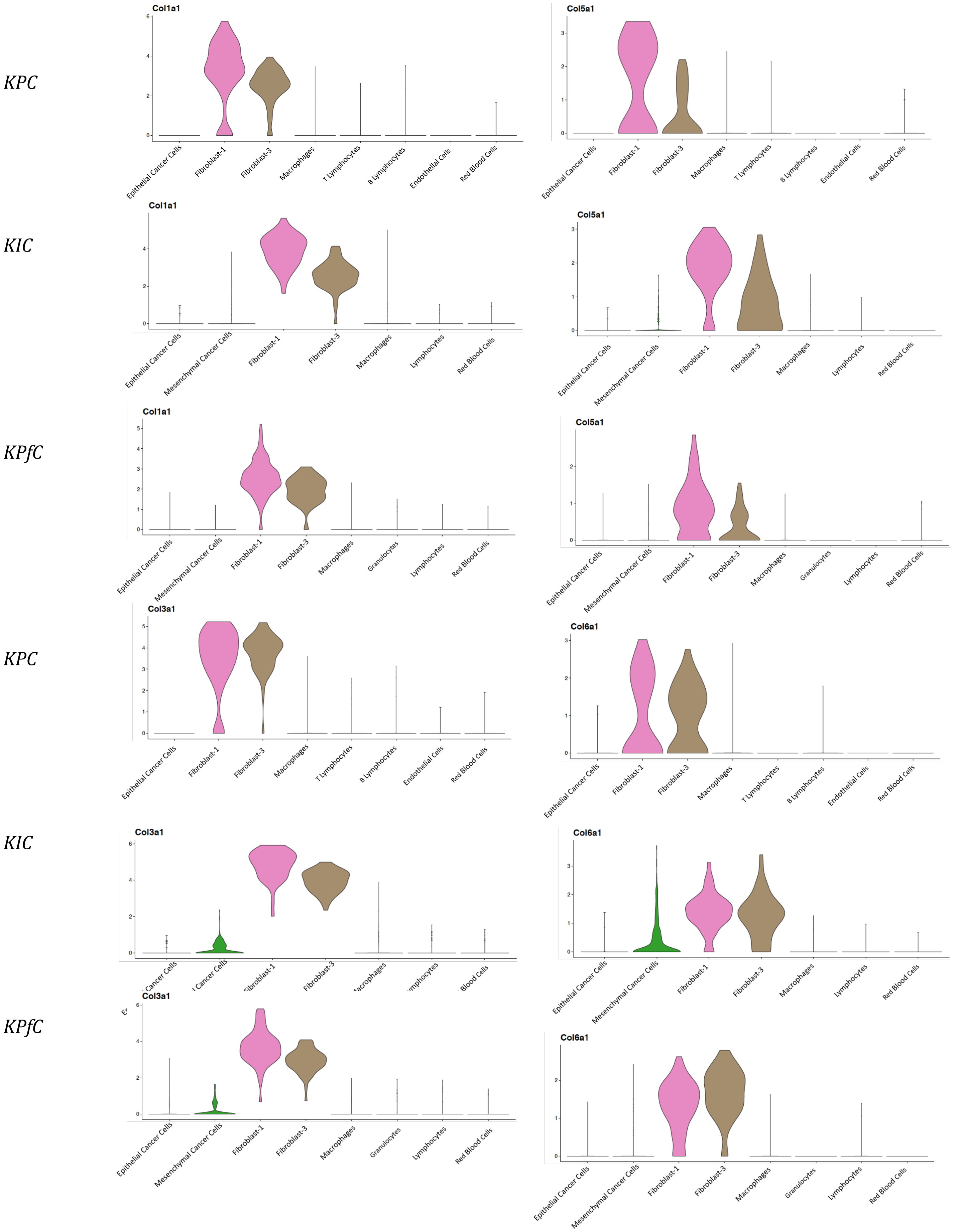
KPC
Col1a1
Epithelial Cancer Cells
Fibroblast-1
Fibroblast-3
Macrophages
T Lymphocytes
B Lymphocytes
Endothelial Cells
Red Blood Cells
Col5a1
KIC
Col1a1
Epithelial Cancer Cells
Mesenchymal Cancer Cells
Fibroblast-1
Fibroblast-3
Macrophages
Lymphocytes
Red Blood Cells
Col5a1
KPfC
Col1a1
Epithelial Cancer Cells
Mesenchymal Cancer Cells
Fibroblast-1
Fibroblast-3
Macrophages
Granulocytes
Lymphocytes
Red Blood Cells
Col5a1
KPC
Col3a1
Col6a1
KIC
Col3a1
Col6a1
KPfC
Col3a1
Col6a1

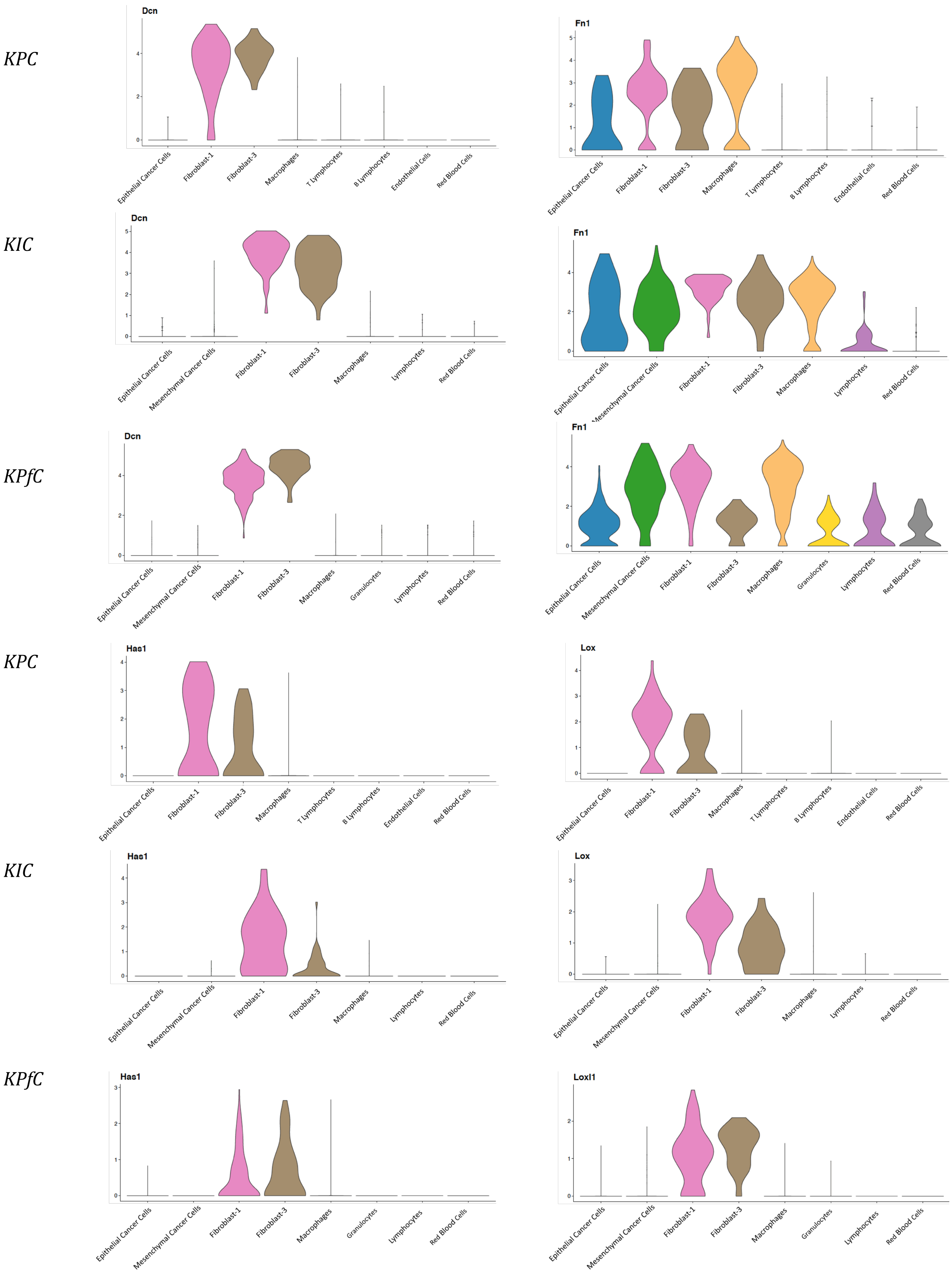
KPC
Dcn
Epithelial Cancer Cells
Fibroblast-1
Fibroblast-3
Macrophages
T Lymphocytes
B Lymphocytes
Endothelial Cells
Red Blood Cells
Fn1
KIC
Dcn
Epithelial Cancer Cells
Mesenchymal Cancer Cells
Fibroblast-1
Fibroblast-3
Macrophages
Lymphocytes
Red Blood Cells
Fn1
KPfC
Dcn
Epithelial Cancer Cells
Mesenchymal Cancer Cells
Fibroblast-1
Fibroblast-3
Macrophages
Granulocytes
Lymphocytes
Red Blood Cells
Fn1
KPC
Has1
Lox
KIC
Has1
Lox
KPfC
Has1
Loxl1

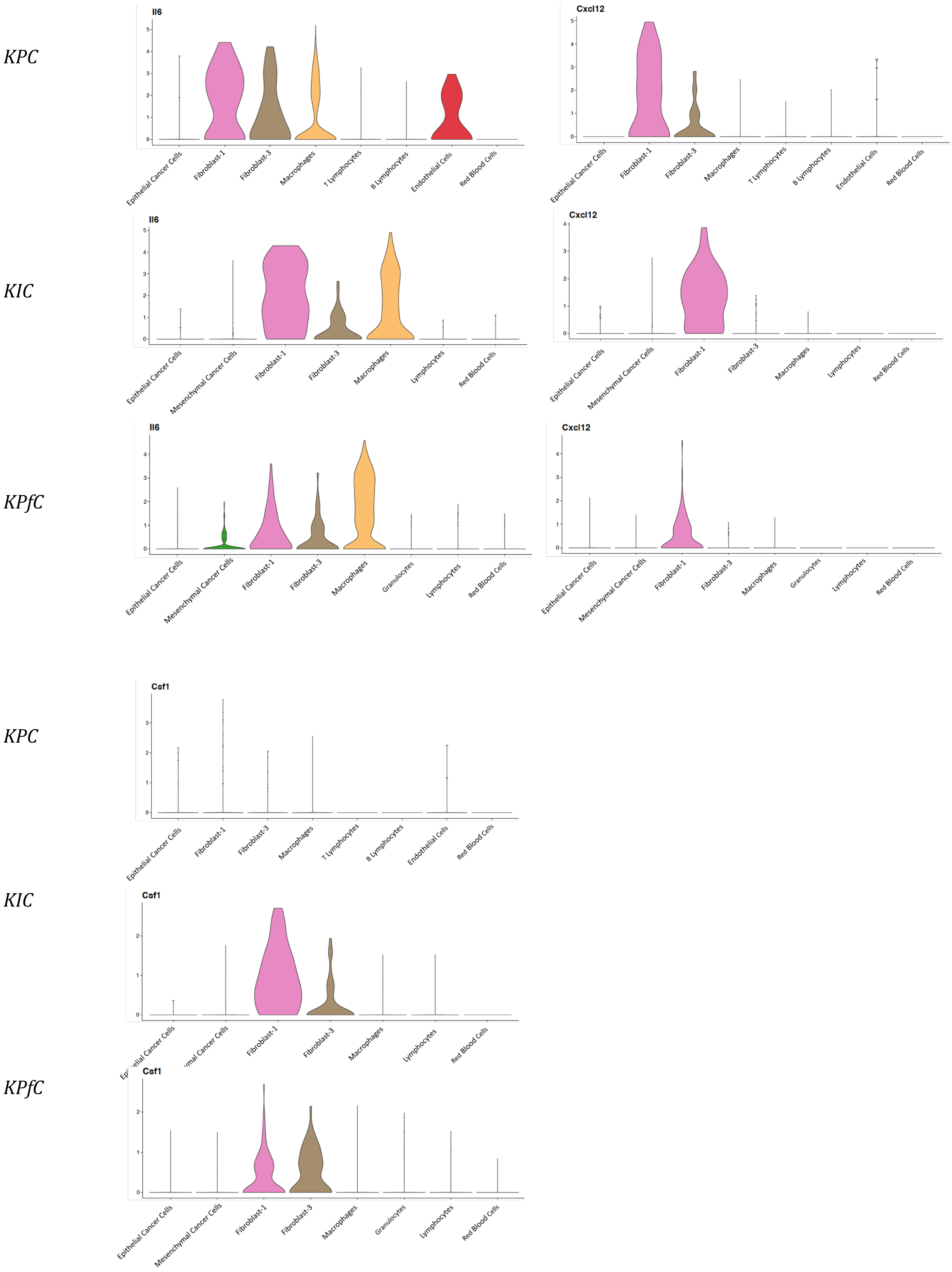

KPC
Il6
Cxcl12
KIC
Il6
Cxcl12
KPfC
Il6
Cxcl12
KPC
Csf1
KIC
Csf1
KPfC
Csf1
Epithelial Cancer Cells
Mesenchymal Cancer Cells
Fibroblast-1
Fibroblast-3
Macrophages
T Lymphocytes
B Lymphocytes
Endothelial Cells
Granulocytes
Lymphocytes
Red Blood Cells

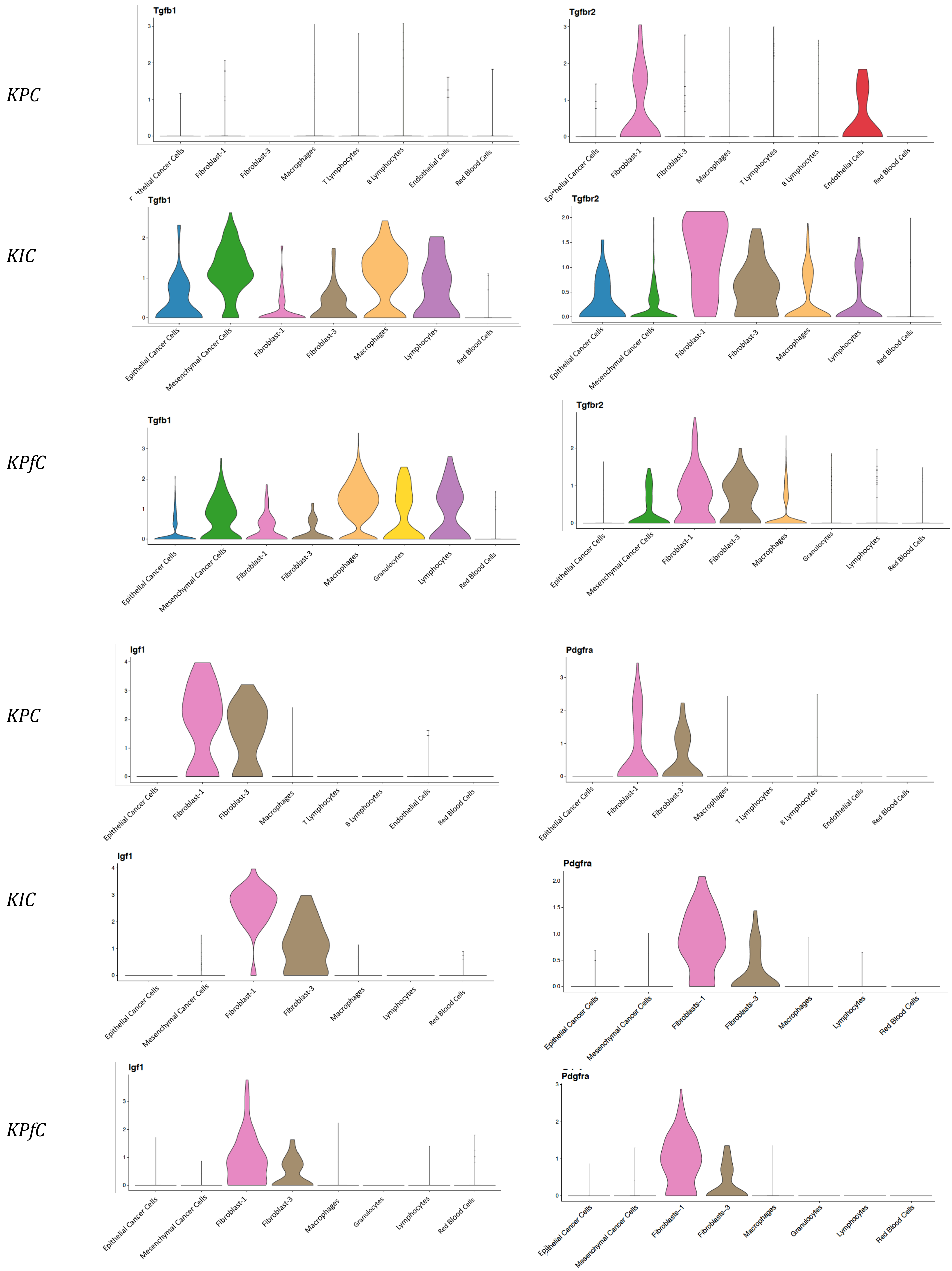

KPC
Tgfb1
Tgfbr2
Epithelial Cancer Cells
Fibroblast-1
Fibroblast-3
Macrophages
T Lymphocytes
B Lymphocytes
Endothelial Cells
Red Blood Cells
KIC
Tgfb1
Tgfbr2
Epithelial Cancer Cells
Mesenchymal Cancer Cells
Fibroblast-1
Fibroblast-3
Macrophages
Lymphocytes
Red Blood Cells
KPfC
Tgfb1
Tgfbr2
Epithelial Cancer Cells
Mesenchymal Cancer Cells
Fibroblast-1
Fibroblast-3
Macrophages
Granulocytes
Lymphocytes
Red Blood Cells
KPC
Igf1
Pdgfra
Epithelial Cancer Cells
Fibroblast-1
Fibroblast-3
Macrophages
T Lymphocytes
B Lymphocytes
Endothelial Cells
Red Blood Cells
KIC
Igf1
Pdgfra
Epithelial Cancer Cells
Mesenchymal Cancer Cells
Fibroblast-1
Fibroblast-3
Fibroblasts-1
Fibroblasts-3
Macrophages
Lymphocytes
Red Blood Cells
KPfC
Igf1
Pdgfra
Epithelial Cancer Cells
Mesenchymal Cancer Cells
Fibroblast-1
Fibroblast-3
Fibroblasts-1
Fibroblasts-3
Macrophages
Granulocytes
Lymphocytes
Red Blood Cells

Supplementary figures 4: Comparison of transcripts for key ECMs, growth factors, and cytokines in iCAF (Fibroblast-1) and myCAFs (Fibroblast-3 versus other tumor cell populations, showing that many key factors are robustly expressed in both myCAFs and iCAFs relative to other tumor cell populations.

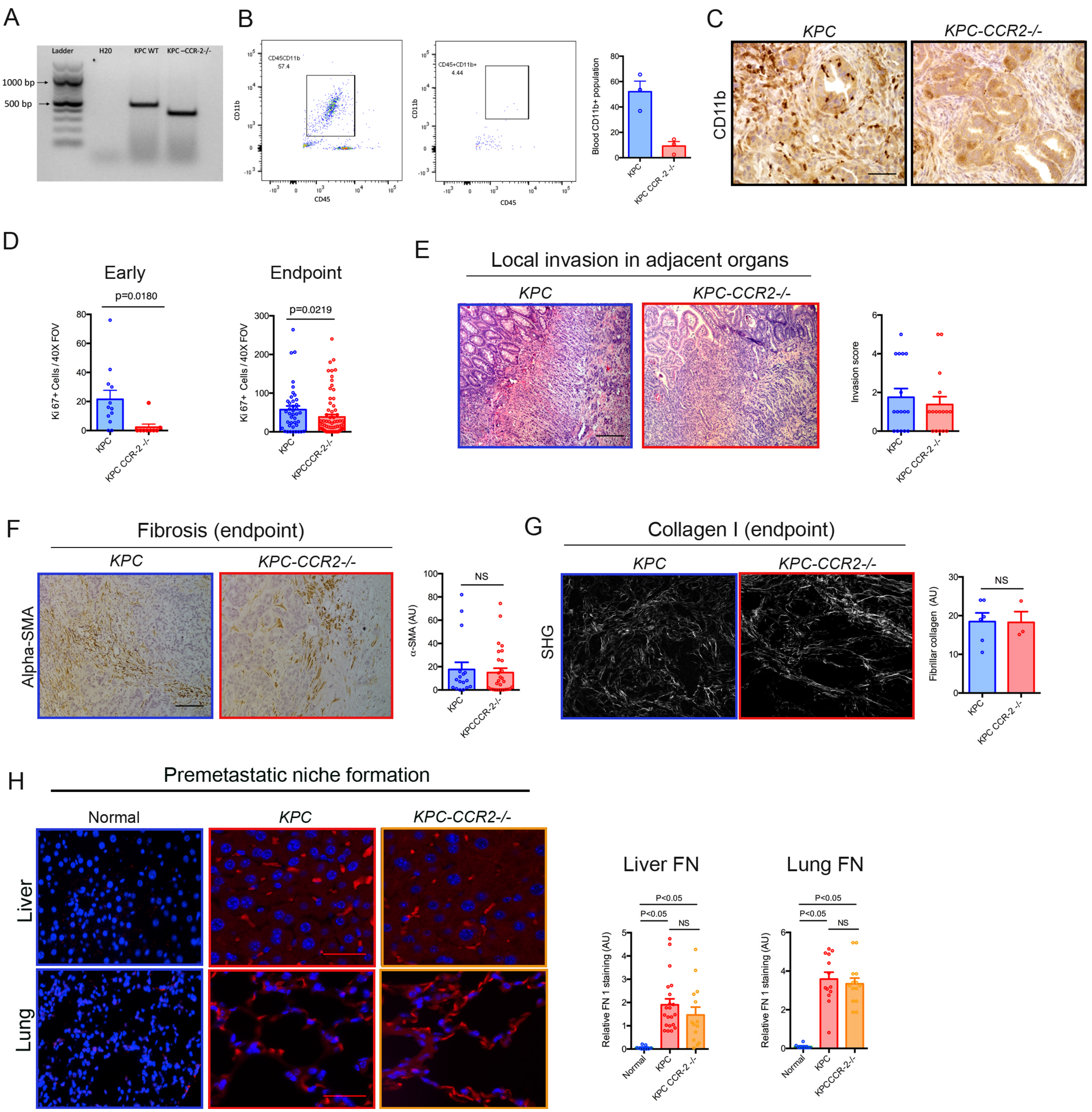

Supplementary figures 5: Genetic deletion of *Ccr2* reduces the disease severity. **A)** Agarose gel electrophoresis showing the amplification of 500 and 550 kb specific for WT *Ccr2* and deleted *Ccr2* gene in mice. **B)** Representative scatter plot of flow cytometry and quantification of CD11b+ cells in *KPC* and *KPC-CCR2*$^{-/-}$ mice showing decreased myeloid cell population in KPC-CCR2$^{-/-}$ mice (n=3mice/group). **C)** IHC of CD11b in diseased pancreata of 8-10-week-old mice showing decrease myeloid cell recruitment. **D)** Quantification of Ki67+ at the early and late stages of PDA in *KPC* and *KPC-CCR2*$^{-/-}$ showing decreased proliferation (n-4-5 mice/group). **E)** H&E images and invasion scores for invasion into adjacent organs showing no change in local invasion. **F)** IHC and quantification of α-SMA in *KPC* and *KPC-CCR2*$^{-/-}$ mice showing no signif9icant difference in CAF levels. **G)** Second harmonic generation imaging and quantification of collagen I in *KPC* and *KPC-CCR2*$^{-/-}$ showing no change in collagen fibrosis. **H)** IF staining and quantitative analysis of fibronectin in the liver and lungs of *KPC* and *KPC-CCR2*$^{-/-}$ mice showing no change in fibronectin content in metastatic sites.

A

Plasma concentration of drug (at 24hrs)

Animal ID	CCR-2i[ng/mL]	Mean [ng/mL]	SD
Ctrl-1	4.4	**4.4**	**0.01**
Ctrl-2	4.4		
KPC-5941	787	**1,214**	**603**
KPC-6309	1,640		

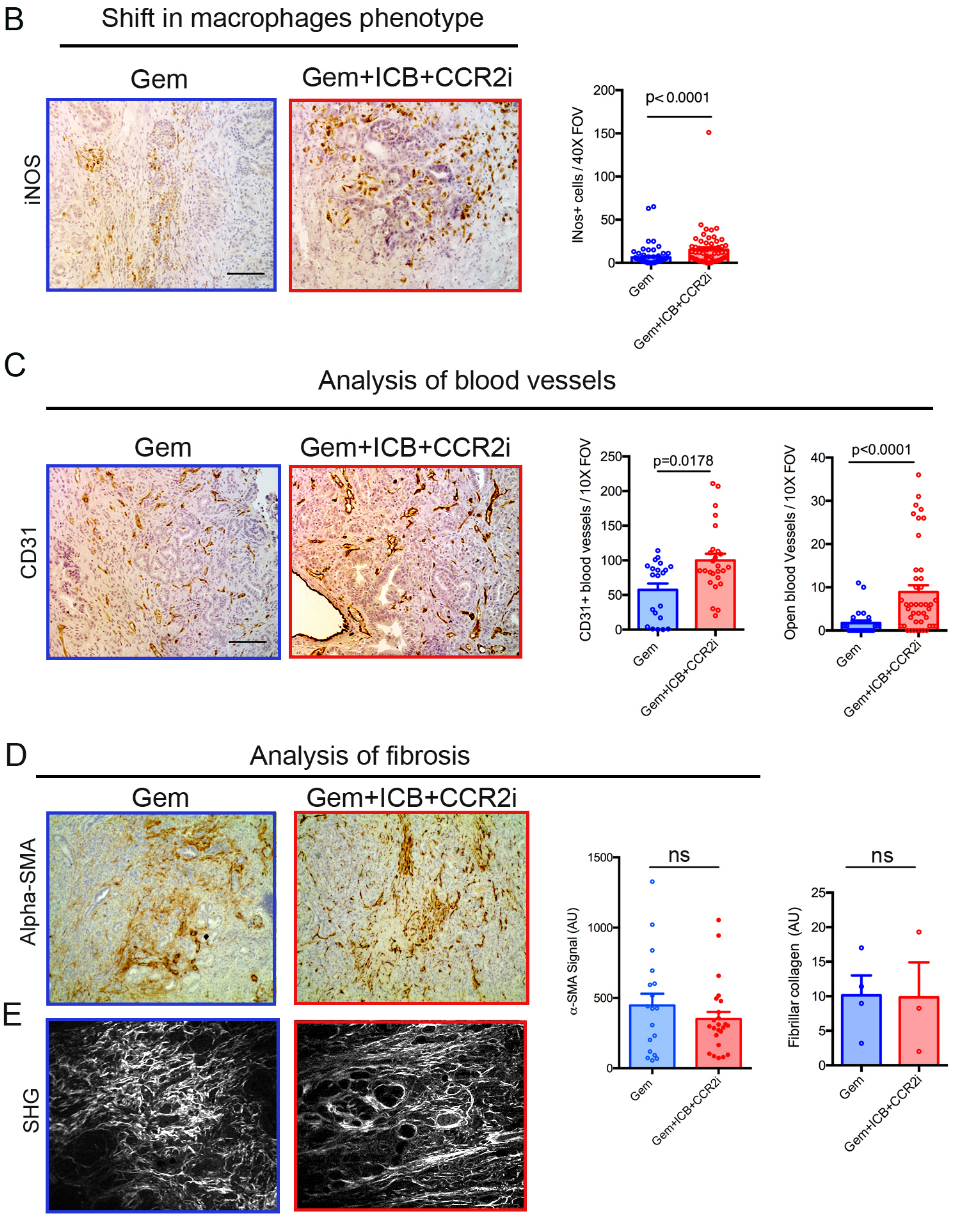

Supplementary figures 6: Blocking TAMs increases responsiveness to immune therapy. A) HPLC analysis showing availability of circulating CCR2 inhibitor in plasma of treated *KPC* mice after 24hrs of treatment. **B)** Gem+ICB+CCR2i results in greater number of pro-tumor polarized macrophages in PDA tumors: IHC and the number of iNOS positive macrophages in Gem, and Gem+ICB+CCR2i treatment groups. **C)** Gem+ICB+CCR2i combination therapy increases micro-vessel density and function (as measured by decreased vascular collapse). **D-E)** Gem+ICB+CCR2i combination therapy does not alter fibroblast activation status or collagen fibrosis.

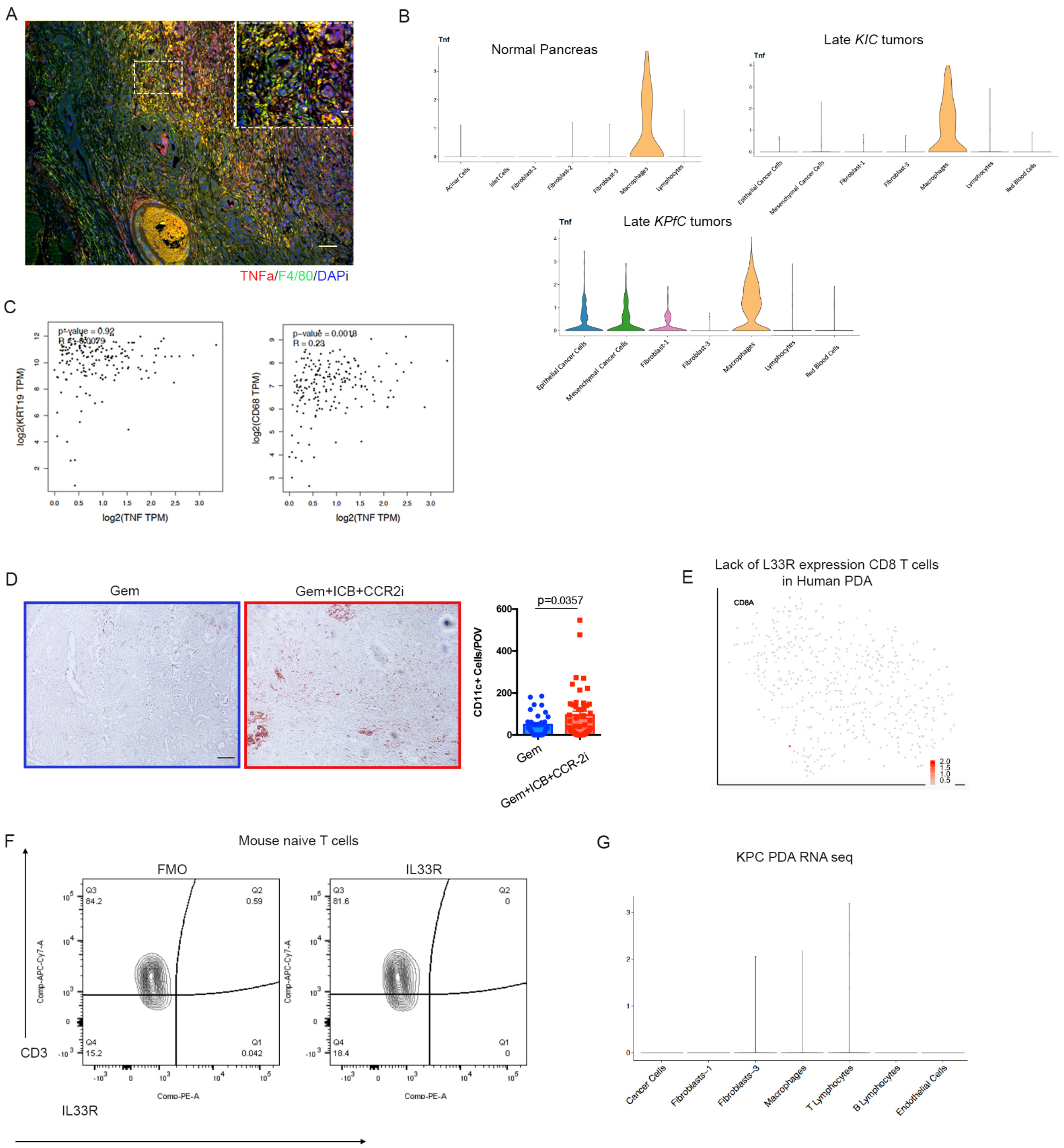

Supplementary figures 7: Macrophages a primary source of TNF-α and T cells lacks IL33R

A) Dual IF staining showing the majority of TNF-α colocalized with F4/80+ macrophages in *KPC* tumors. **B)** Violin plot shows high expression of TNF-α in macrophages in normal and *KIC* and *KPfC* tumors. **C)** Strong to moderate correlations between IL33 and markers of CD8 T cell functionality (TCGA dataset analysis). **D)** IHC and quantification of CD11c in Gem and Gem+ICB+CCR-2i group. **E)** Human PDA single cell RNAseq scattered plot shows lack of IL33R positive CD8+ T cells. **E)** t-sne plot showing lack of IL33 receptors on human CD8 T cells. **F)** Flow cytometry analysis shows lack of IL33R on naïve T cells from healthy mouse spleen. **G)** Single cell RNAseq from *KPC* tumors shows low expression of IL33 receptors in various PDA immune cells.

Supplemental Tables:

Supp. Table S1 Clinical Table with metastatic burden in *KPC* and *KPC-CCR2*$^{-/-}$
Supp. Table S2 Clinical Table with metastatic burden in the treatment cohorts
Supp. Table S3 List of primers used in this study
Supp. Table S4 List of antibodies used in this study
